# Genome-wide nucleosome footprints of plasma cfDNA predict preterm birth: A case-control study

**DOI:** 10.1371/journal.pmed.1004571

**Published:** 2025-04-15

**Authors:** Zhiwei Guo, Ke Wang, Xiang Huang, Kun Li, Guojun Ouyang, Xu Yang, Jiayu Tan, Haihong Shi, Liangping Luo, Min Zhang, Bowei Han, Xiangming Zhai, Jinhai Deng, Richard Beatson, Yingsong Wu, Fang Yang, Xuexi Yang, Jia Tang

**Affiliations:** 1 Department of Obstetrics and Gynaecology, Guangzhou First People’s Hospital, School of Medicine, South China University of Technology, Guangzhou, China; 2 NHC Key Laboratory of Male Reproduction and Genetics, Guangdong Provincial Reproductive Science Institute (Guangdong Provincial Fertility Hospital), Guangzhou, China; 3 Institute of Antibody Engineering, School of Laboratory Medicine and Biotechnology, Southern Medical University, Guangzhou, China; 4 Department of Obstetrics and Gynaecology, Nanfang Hospital, Southern Medical University, Guangzhou, China; 5 Prenatal Diagnosis Center, Foshan Women and Children Hospital, Foshan, Guangdong, China; 6 Guangzhou Darui Biotechnology Co, Ltd., Guangzhou, China; 7 Emergency Department, Foshan Women and Children Hospital, Foshan, Guangdong, China; 8 Medical Genetics Center, Jiangmen Maternity and Child Health Care Hospital, Jiangmen, Guangdong, China; 9 School of Medicine, Jinan University, Guangzhou, China; 10 Richard Dimbleby Department of Cancer Research, Comprehensive Cancer Centre, Kings College London, London, United Kingdom; 11 King’s College London, School of Cancer and Pharmaceutical Sciences, Guy’s Cancer Centre, London, United Kingdom; 12 Department of Fetal Medicine and Prenatal Diagnosis, Zhujiang Hospital, Southern Medical University, Guangzhou, China; 13 Department of Molecular and Human Genetics, Baylor College of Medicine, Houston, Texas, United States of America; King's College, UNITED KINGDOM OF GREAT BRITAIN AND NORTHERN IRELAND

## Abstract

**Background:**

Preterm birth (PTB) occurs in approximately 11% of all births worldwide, resulting in significant morbidity and mortality for both mothers and their offspring. Identifying pregnancies at risk of preterm birth during early pregnancy may help improve interventions and reduce its incidence. Plasma cell-free DNA (cfDNA), derived from placenta and other maternal tissues, serves as a dynamic indicator of biological processes and pathological changes in pregnancy. These properties establish cfDNA as a valuable biomarker for investigating pregnancy complications, including PTB.

**Methods and findings:**

To date, there are few methods available for PTB prediction that have been developed with large sample sizes, high-throughput screening, and validated in independent cohorts. To address this gap, we established a large-scale, multi-center case-control study involving 2,590 pregnancies (2,072 full-term and 518 preterm) from three independent hospitals to develop a spontaneous preterm birth classifier. We performed whole-genome sequencing on cfDNA, focusing on promoter profiling (read depth of promoter regions spanning from −1 to +1 kb around transcriptional start sites). Using four machine learning models and two feature selection algorithms, we developed classifiers for predicting preterm birth. Among these, the classifier based on the support vector machine model, named PTerm (**P**romoter profiling classifier for pre**term** prediction), exhibited the highest area under the curve (AUC) value of 0.878 (0.852–0.904) following leave-one-out cross-validation. Additionally, PTerm exhibited strong performance in three independent validation cohorts, achieving an overall AUC of 0.849 (0.831–0.866).

**Conclusions:**

In summary, PTerm demonstrated high accuracy in predicting preterm birth. Additionally, it can be utilized with current non-invasive prenatal test data without changing its procedures or increasing detection cost, making it easily adaptable for preclinical tests.

## Author summary

**Why was this study done?:**

- Preterm birth (PTB) is a common pregnancy complication affecting approximately 11.1% of newborns worldwide.
- However, reliable biomarkers for pregnancy complications remain scarce, making the identification of PTB biomarkers an urgent priority.
- In this study, we hypothesize that the nucleosome profiles of plasma cell-free DNA (cfDNA) carry information about its originating tissues, which could be used to develop predictive methods for preterm birth. To validate its potential for preterm birth prediction in early gestation, we conducted a large-scale, retrospective study.

**What did the researchers do and find?:**

- We analyzed cfDNA promoter profiling from NIPT data to identify differences between pre- and full-term pregnancies. Our findings demonstrated a significant association between cfDNA promoter profiling and preterm birth.
- To further assess its predictive potential, we utilized NIPT data from 2,590 pregnancies across three independent hospitals to develop a robust predictive classifier for preterm birth. This classifier, termed PTerm, exhibited strong predictive performance, achieving an overall AUC of 0.849 (0.831–0.866).
- PTerm holds promise as a tool for the early identification of pregnancies at risk of preterm birth.

**What do these findings mean?:**

- Our data suggest that the promoter-profiling-based classifier (PTerm) could provide valuable PTB predictions in early pregnancy.
- Our method is also easily applicable to routine NIPT data and does not require any additional tests or increase detection costs, making it feasible in clinical practice.
- Given this, we believe that our method serves as a critical stepping stone toward developing a non-invasive diagnostic for the early prediction of pregnancy complications.

## Abstract 3

In a case-control study, Zhiwei Guo and colleagues investigate the use of nucleosome profiles of plasma cfDNA to predict preterm birth.

## Introduction

Preterm birth (PTB) is a common pregnancy complication affecting in approximately 11.1% of newborns worldwide [1]. Additionally, PTB is a major determinant of infant morbidity and mortality, responsible for approximately 35% of pregnancy-related deaths, and it leads to adverse maternal and fetal outcomes, including increased long-term risks of motor, cognitive, and behavioral disorders [2]. The development of interventions critically depends on the early identification of pregnant women at risk of PTB. Given that the maternal circulatory system carries both maternal and fetal information, multivariate screening methods based on maternal blood omics data, such as metabolites and cell-free RNA (cfRNA) [3–5], have recently been proposed. However, reliable biomarkers for pregnancy complications remain scarce, making the identification of PTB biomarkers an urgent priority.

Plasma cell-free DNA (cfDNA) has broad application in various clinical settings, owing to its remarkable stability and practicality for routine clinical applications [6,7]. In pregnancies, cell-free fetal DNA (cffDNA) mainly originates from the placenta and represents the genetic material of the fetus. The presence of cffDNA within maternal cfDNA facilitates non-invasive prenatal testing (NIPT), allowing for the screening of fetal chromosomal abnormalities, such as trisomies 21, 18, and 13, as well as sex chromosome aneuploidies [7]. Although NIPT has been widely, its application has primarily focused on screening a limited number of diseases based on the distribution characteristics of cfDNA. Recently, other disease-related features of cfDNA, such as terminal motifs and promoter profiles, have been identified and utilized for disease screening. However, their associations with PTB remain unclear. Therefore, it is urgent to identify new disease-specific characteristics of cfDNA to expand the application of NIPT in addressing pregnancy complications, especially premature delivery in early pregnancy.

During pregnancy, plasma cfDNA comprises fragmented DNA released from placental trophoblasts and hematopoietic cells during their apoptosis via enzymatic chromatin process. Exposed DNA between nucleosomes is degraded by apoptotic nucleases, while nucleosome-bound DNA remains preserved [8,9]. Nucleosomes are densely positioned around gene regulatory regions, such as promoters and enhancers. Consequently, genes with varying expression levels exhibit distinct nucleosome profiles at gene promoters, which can be used to infer the expression profiles of placental trophoblasts and hematopoietic cells [8,10]. For instance, a higher read depth leads to more coverage in pTSS, resulting in a larger nucleosomal footprint at the promoter and decreased gene expression. PTB commonly arises as a complication of placental dysfunction and alterations in the maternal immune system [11]. Placental dysfunction can lead to serious complications during pregnancy, ultimately resulting in PTB [11]. Furthermore, abnormalities in immune cell functions, such as imbalances in immune cell subsets, excessive activation of inflammatory reactions, and disruption of immune regulatory pathways, can increase the risk of premature birth [12,13]. Therefore, the nucleosome profile of plasma cfDNA is closely related to PTB.

In this study, we hypothesize that the nucleosome profiles of plasma cfDNA carry information about its originating tissues, which could be used to develop predictive methods for PTB. To validate its potential for PTB prediction in early gestation, we conducted a large-scale, retrospective study. Currently, NIPT is performed in more than 60 countries, with over 10 million tests conducted each year. Our method relies on NIPT data without altering its procedure or increasing detection costs, making it easily adaptable for preclinical tests. Therefore, our findings highlight the potential of Promoter profiling classifier for preterm prediction (PTerm), which relies on genome-wide promoter profiling of plasma cfDNA, as a simple and precise method for identifying pregnancies at risk of PTB.

## Methods

### Participant characteristics

In total, we collected 2,590 plasma samples from pre- and full-term pregnancies. Plasma samples were collected once per pregnant participants, between 12 and 28 weeks of gestation from three independent hospitals in China: Jiangmen maternal and child healthcare hospital (JM), Foshan maternal and child healthcare hospital (FS), and Nanfang hospital of southern medical university (NFY). Of the 2,590 participants, 518 women experienced a preterm delivery, while the remaining 2,072 women delivered at full-term (Table 1). Participants from JM were collected between January 1, 2017 and December 30, 2020. Participants enrolled at FS were recruited between December 1, 2018 and December 1, 2020. Participants from NFY were enrolled between May 1, 2016 and May 1, 2020. The institutional ethics committees of all hospitals approved this retrospective analysis, and informed written consent was obtained from all participants (IRB number: 2019053).

**Table 1:** Clinical characteristics of pregnancies in four cohorts.

	Training cohort (*n* = 915)	Validation cohort1 (*n* = 395)	Validation cohort2 (*n* = 930)	Validation cohort3 (*n* = 350)	*P*-value
Full-term pregnancies					
*n*	732	316	744	280	
Gestational age at sampling (weeks)	15.1 ± 3.2	15.3 ± 3.3	15.5 ± 3.5	15.5 ± 3.7	0.353^w^
Gestational age of birth (weeks)	39.7 ± 0.8	39.7 ± 0.8	39.6 ± 0.8	39.6 ± 0.8	0.264^w^
Maternal age (years)	29.8 ± 4.7	30.3 ± 4.6	30.5 ± 4.1	30.5 ± 4.6	0.056^w^
BMI (kg/m^2^)	20.8 ± 2.8	20.6 ± 2.9	20.6 ± 1.1	20.6 ± 2.7	0.479^w^
Birth length (cm)	49.1 ± 1.5	49.1 ± 1.4	49.2 ± 0.6	49.1 ± 1.4	0.491^w^
Birth weight (kg)	3.2 + 0.3	3.2 ± 0.3	3.2 ± 0.1	3.2 ± 0.3	0.100^w^
Previous birth, No. (%)					0.099^^p^^
0	312 (42.6%)	147 (46.5%)	329 (44.2%)	128 (45.7%)	
1	392 (53.6%)	161 (50.9%)	370 (49.7%)	143 (51.1%)	
≥2	28 (3.8%)	8 (2.6%)	45 (6.1%)	9 (3.2%)	
Preterm pregnancies					
n	183	79	186	70	
Gestational age at sampling (weeks)	15.1 ± 3.2	15.3 ± 3.3	15.5 ± 3.8	15.5 ± 3.5	0.803^w^
Gestational age of birth (weeks)	35.3 ± 1.8	35.2 ± 1.7	34.9 ± 2.1	34.7 ± 2.6	0.066^w^
Maternal age (years)	30.7 ± 5.1	30.1 ± 4.8	30.8 ± 4.9	30.5 ± 4.8	0.797^w^
BMI (kg/m^2^)	21.2 ± 3.1	21.1 ± 3.0	20.7 ± 2.6	20.6 ± 1.2	0.619^w^
Birth length (cm)	46 ± 2.7	45.9 ± 3.0	46.2 ± 2.4	46.1 ± 1.6	0.345^w^
Birth weight (kg)	2.46 ± 0.51	2.42 ± 0.47	2.46 ± 0.56	2.47 ± 0.37	0.768^w^
Previous birth, No. (%)					0.069^^f^^
0	82 (44.8%)	32 (40.5%)	96 (51.6%)	29 (41.4%)	
1	94 (51.4%)	43 (54.4%)	73 (39.2%)	35 (50.0%)	
≥2	7 (3.2%)	4 (5.1%)	17 (9.2%)	6 (8.6%)	
Type of preterm birth					
Very preterm	13 (7.1%)	7 (8.9%)	26 (14.0%)	6 (8.6%)	
Moderate preterm	16 (8.5%)	7 (8.9%)	20 (10.8%)	9 (12.9%)	
Late preterm	154 (84.2%)	65 (82.2%)	140 (75.2%)	55 (78.5)	

Data are presented as mean ± standard deviation. BMI = pre-pregnancy body mass index. Two-sided Wilcoxon rank-sum test (^w^) was used for the comparison of continuous variables (*n* = 915 for training cohort; 390 for validation cohort 1; 930 for validation cohort 2; 350 for validation cohort 3). Two-sided Pearson *χ*2 test (^p^) and Fisher exact test (^f^) were used for the comparison of categorical variables (*n* = 915 for training cohort; 390 for validation cohort 1; 930 for validation cohort 2; 350 for validation cohort 3).

Pregnancies were excluded if they met any of the following criteria: (1) Multiple pregnancies; (2) Uterine fibroids or malformation; (3) Chorioamnionitis; (4) Chromosomal or congenital abnormalities; (5) Pregnancies with assisted reproductive technology. After exclusion, samples were retrospectively assigned to a birth outcome group based on their subsequent delivery time, with spontaneous premature delivery defined as birth before 37 weeks of gestation. Gestational age was determined based on the last menstrual period and ultrasonography. PTB was classified as spontaneous if a woman presented with either cervical dilation and/or preterm premature rupture of membranes (PPROM) and delivered before 37 weeks of gestation. Spontaneous labor with intact membranes accounted for 53.1% of spontaneous preterm birth (sPTB), while PPROM accounted for 46.9%. Delivered for maternal indications (e.g., preeclampsia) or fetal indication (e.g., fetal distress) were excluded from the sPTB category. Full-term controls included pregnancies with a gestational age exceeding 38 weeks, excluding those with pregnancy complications. Following the case-control ratios from previous case-control studies [14–16], four full-term controls were selected for each preterm pregnant case, matched by maternal age, gestational age at sampling, and body mass index (BMI). The clinical characteristics of preterm pregnancies and their corresponding controls were well matched across four cohorts (Table 1; all *P*-value >  0.05, Mann–Whiney U test).

### DNA-Seq processing and promoter profiling analysis

The procedure for sample collection, cfDNA isolation, and DNA sequencing are detailed in the S1 Text. We estimated the fetal fraction using the proportion of all sequencing reads from the Y chromosome or the seqFF method [17]. Gene information was obtained from the RefSeq of the University of California Santa Cruz Genome Browser Database [18]. For each transcript, the promoter region, spanning from −1 to +1 kb around the transcriptional start site, was defined as pTSS regions. pTSS regions overlapping with the Duke blacklist region were removed (http://hgdownload.cse.ucsc.edu/goldenpath/hg19/encodeDCC/wgEncodeMapability/). After sequencing, the raw reads were aligned to the human reference genome (hg19) using bwa-mem (ver. 0.7.4). Polymerase chain reaction (PCR) duplicates were removed using the rmdup function of SAMtools (ver. 1.2). GC-bias correction was implemented using deeptools (ver. 3.5.0) with default settings. Read coverage for each pTSS region was extracted using bedtools (ver. 2.17.0). We normalized the read coverage data using the following formula:

Since the pTSS region was defined as −1 to +1 kb around the TSS, the pTSS length of each gene is equivalent to 2,000. Read depth refers to the number of cfDNA reads overlapping specific genomic regions. cfDNA coverage around TSS refers to the reads of cfDNA overlapping with the pTSS region, which reflects the extent of nucleosomal footprints at the promoter.

### Gene expression profile analysis and gene information acquisition

Placenta and whole blood expression profiles for preterm pregnancies (GSE73685) [19] were downloaded from the Gene Expression Omnibus (GEO) database. Additional placenta (GSE148402 [20] and GSE174415 [21]) and whole blood expression profiles of preterm pregnancies (GSE46510 [20] and GSE59491 [22]) were also downloaded from GEO and normalized using GEOquery (ver. 3.3.1). The top 500 highly expressed and 500 least expressed genes, representing the highest and lowest mean expression levels in the placenta and whole blood, were identified by analyzing their expression profiles in GSE73685 (S1 Table). Placenta- and blood-enriched genes were obtained from the study by Gong and colleagues [23] and PaGenBase [24], respectively (S2 Table). Genes with expression levels below 0.1 in all tissues according to FANTOM5 were defined as unexpressed genes. Housekeeping genes, defined as those widely expressed across various tissues, along with unexpressed genes were obtained from previous studies (S3 Table) [9].

### Genes with significant differential promoter coverages

At the discovery stage, we selected 20 PTB cases and 20 full-term pregnancies (S4 Table) and then performed whole-genome sequencing of their cfDNA. After data processing and normalization, pTSS coverages between pre- and full-term samples were compared to calculate the *P*-value using the Wilcoxon rank-sum test. The raw *P-*values were adjusted to the false discovery rate (FDR) using the Benjamini–Hochberg procedure. Genes with significant differential coverages in the pTSS regions were identified by log_2_ | fold change | ≥  1 and FDR ≤  0.05 (S5 Table).

### Sample clustering and gene function annotation

Principal component analysis (PCA) was performed using the prcomp function of the stats package, and then the results were plotted using the ggord package (ver. 1.1.7). Hierarchical clustering of the coverage data with complete linkage clustering algorithms was implemented using the pheatmap package (ver. 1.0.2). Enrichment analysis of gene ontology (GO) was completed using Metascape (ver. 20220101) [25], and Kyoto Encyclopedia of Genes and Genomes (KEGG) was completed using clusterProfiler (ver. 3.18.1) [26] with their default settings.

### Gene correlation network construction

To construct the gene correlation network with genes exhibiting differential coverages, functional relationships were obtained from the String database (ver. 11.5) [27], which served as a reference for known protein-protein interactions and guided the connections displayed in the network. The network was then visualized with Cytoscape (ver. 3.8), which depicted gene interactions by illustrating interactions according to correlation strengths. Cytohubba (ver. 0.1) [28] was then used to assess centrality and importance within the network, aiding in the identification of hub genes. Finally, 10 genes with the highest gene degrees as the top 10 hub genes were selected.

### Predictive classifier construction and validation

To develop classifiers for predicting spontaneous PTB, we preformed whole-genome sequencing of cfDNA on 2,590 pregnant women, including 518 preterm and 2,072 full-term pregnancies from three independent hospitals: JM, FS, and NFY. The 20 preterm and 20 full-term pregnancies used in the discovery stage were randomly selected from JM, with well-matched gestational age, maternal age, and BMI (S4 Table). These samples were also included in the training stage. Samples collected from JM (*n* = 1,310) were randomly divided into training (*n* = 915, training cohort [*n* = 915]: 183 cases and 732 controls) and internal validation cohorts (validation1 cohort [*n* = 395]: 79 cases and 316 controls) at a ratio of 7:3. Samples from FS (validation2 cohort [*n* = 930]: 186 cases and 744 controls) and NFY (validation3 cohort [*n* = 350]: 70 cases and 280 controls) were used as external validation cohorts. The clinical characteristics of the pre- and full-term pregnancies were well-matched across the four cohorts (Table 1). The workflow for classifier construction is illustrated in S1 and S2 Figs. Since many studies have reported that discrete data may improve the predictive performance [29–31], we discretized the read coverage of each pTSS identified in the discovery cohort according to the optimal cut-off point with the largest AUC value in the training cohort before building the classifiers. A total of 277 genes exhibiting differential coverages were subsequently used for classifier construction. The detailed workflow of classifier construction has been illustrated S1 Fig. Previous studies have demonstrated that discretization enhances data interpretability, uncovers non-linear relationships, harmonizes mixed-type datasets, and facilitates the derivation of count data from continuous variables [32]. Therefore, the read coverage for each promoter in each subject was set to one when it was larger than the corresponding optimal cut-off; otherwise, it was set to zero. Then, the sigFeature package (ver. 1.8.0) was used to evaluate the importance of the pTSS regions [33]. Pearson correlation coefficients were calculated for all pairs of genes with differential coverages. Highly correlated genes with higher importance were retained (S5 Table, | *r* | >  0.5). To minimize collinearity in the model predictors, 49 highly correlated variables were removed, resulting in a final set of 228 genes for further analysis (S5 and S6 Tables).

We then evaluated four predictive models, including support vector machine (SVM), logistic regression (LR), random forest (RF), and XGBoost (XGB) as the basis for developing a novel predictive classifier for PTB, referring to published studies [29,34]. The SVM model was constructed using the linear kernel in the e1071 package (ver. 1.7.9) with default settings. The LR model was developed using the glm functions of the stats package. The RF model was constructed using the randomForest package. The XGBoost was constructed using the xgboost package. The grid search method was employed to determine the optimal hyperparameters for the random forest and XGBoost models. Each of these predictive models was implemented independently using both backward and lasso algorithms for feature selection. For backward-like feature selection, one feature was deleted at a time, and the classifier with the maximum AUC after 10-fold cross-validation (CV) among all classifiers was selected. The feature to be deleted was chosen according to the maximum AUC increase after deletion. For lasso feature selection, the best lambda was identified using 10-fold CV with the cv.glmnet function from glmnet package (ver. 4.1), using parameters: type.measure = “AUC” and family = “binomial”, and then the features with non-zero coefficients were selected. These selected features were independently used as inputs for the LR, SVM, random forest, and XGBoost models to develop classifiers for PTB prediction. The robustness of the trained classifiers was assessed using the leave-one-out cross-validation (LOOCV) method. Briefly, each subject in the training cohort was withheld in turn, and the remaining subjects were used to train the classifier. The trained classifier was then used to predict the class of the withheld subject. This process continued until all subjects in the training cohort had been judged. Based on the AUC after LOOCV, the classifier with the highest AUC, named PTerm, was selected for further validation. PTerm is based on SVM model with the backward feature selection algorithm, ultimately including 83 genes (S7 Table).

To further assess the performance of PTerm, whole-genome sequencing data from one internal validation (JM, validation1 cohort) and two external validation cohorts (FS, validation2 cohort; NFY, validation3 cohort) were used. The performance of PTerm was further evaluated using data from these internal and external cohorts.

### Statistical analysis

The power calculation of the sample size in the discovery stage was around 80% with 20 premature delivery and 20 full-term pregnancies (S1 Text). Two-sided Wilcoxon rank-sum test was used to compare the continuous variables between the pre- and full-term groups, while two-sided Pearson’s *χ*^2^ and Fisher’s exact tests were used for comparisons of categorical variables. Clinical data was presented as mean ±  standard deviation (SD). The Wilcoxon rank-sum test was used to identify genes with differential read coverages within the pTSS regions and *P*-values of <  0.05 in two-sided tests were considered to be statistically significant. The mean coverage levels of the top 500 highly expressed and the bottom 500 least expressed genes were first calculated for each individual in preterm pregnancies. Subsequently, the differences in coverage between these two groups of genes were analyzed using the Wilcox Signed-Rank test. A similar approach was applied to evaluate the coverage differences between housekeeping genes and unexpressed genes. The R software (ver.4.2) was used for statistical analysis. ROC curves and the significant differences in their AUC, sensitivity, and specificity were plotted and calculated using the pROC package in R.

## Results

### cfDNA carries information about its origin in pregnant women

**Fig 1:**
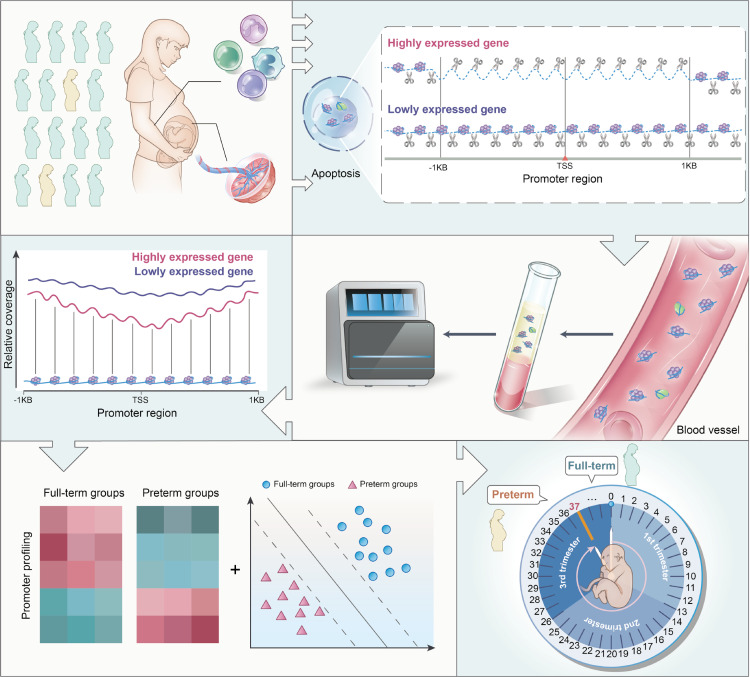
Schematic overview of predicting preterm birth using promoter profiling of plasma cfDNA. During pregnancy, plasma cell-free DNA (cfDNA) is mainly derived from placental trophoblasts and maternal hematopoietic cells, released by their apoptotic cells. Exposed DNA not bound to nucleosomes is digested, while nucleosome-bound DNA escapes digestion and enters into the maternal circulation. Through whole-genome sequencing, we found that read coverages at pTSS regions (−1 to +1 kb around the transcription start site [TSS]) reflect the gene expression patterns of their tissues of origin. Since premature delivery is closely associated with dysfunction and changes in the placenta and maternal immune system, we proposed that cfDNA coverages at pTSS regions could predict preterm birth at early gestational ages. We tested this hypothesis using high-throughput whole-genome sequencing of plasma cfDNA from 2,590 pre- and full-term pregnancies across three independent hospitals. By comparing their promoter profiling, we found differences between pre- and full-term pregnancies. We then used genes with differential promoter coverage and four machine-learning models to develop predictive classifiers for preterm birth. Nucleosome-depleted regions are typically found upstream of the TSS. To show greater differences, all nucleosomes in the promoter regions of highly expressed genes are depicted as depleted in the figure.

Previous studies have reported that cfDNA carries information regarding its tissues of origin [8–10,35], making it suitable for evaluating PTB. Thus, we designed experiments to characterize the cfDNA profiles of pregnancies resulting in pre- and full-term births (Fig 1). To this end, we collected whole-genome sequencing data from 20 preterm and 20 full-term pregnancies (S4 Table). Additionally, we collected the RNA expression profiles of both the placenta and whole blood from preterm pregnancies (GSE73685) [19].

**Fig 2:**
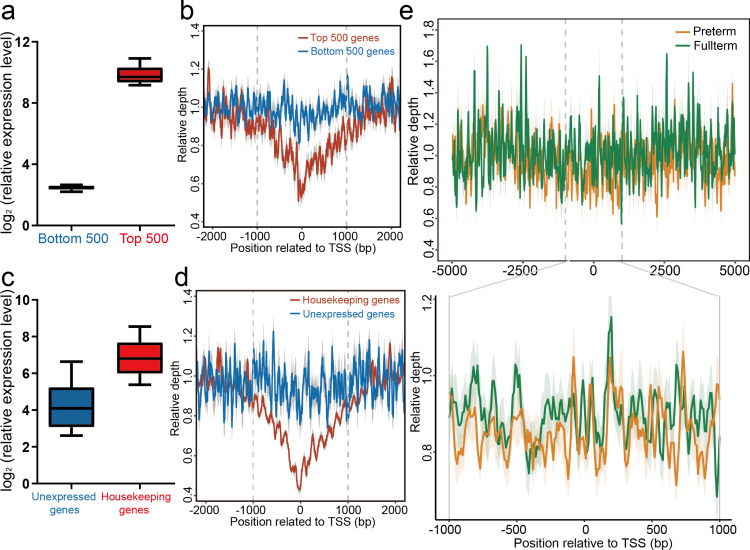
cfDNA profiles of promoter regions reflect nucleosome positioning in pregnant women. ( **a)** Average expression of the 500 most- (Top500, red) and least-expressed (Bottom500, blue) genes in the placenta of preterm birth pregnancies. **(b)** Read depth of whole-genome sequencing across pTSS regions (spanning from −1 to +1 kb around TSS) for the 500 most- (Top500, red line) and least-expressed (Bottom500, blue line) genes. The read depth of the Top500 genes was lower than that of the Bottom500 genes (*P*-value =  1.9e−06, Wilcoxon Signed-Rank test). **(c)** Average expression levels of housekeeping (red) and unexpressed (blue) genes in the placenta. **(d)** Read depth of whole-genome sequencing at the pTSS region of the housekeeping genes (red line) was lower than that of unexpressed genes (blue line) in the placenta (*P*-value =  1.9e−06, Wilcoxon Signed-Rank test, including 2,985 housekeeping and 670 unexpressed genes). **(e)** Sequencing read depth of placenta-enriched genes was more depleted in preterm (yellow line) pregnancies than in full-term (green line) pregnancies (*P*-value =  7.386e−11, Wilcoxon rank-sum test). The RNA expression profiles of the placenta from preterm pregnancies were downloaded from GEO (GSE73685). For the boxplot, the center line represents the median of the data distribution. The box limits denote the interquartile range (IQR), with the lower and upper edges corresponding to the first (Q1) and third quartiles (Q3), respectively. The whiskers extend to the smallest and largest values within 1.5 times the IQR from Q1 and Q3. The Top500 and bottom500 genes are based on the RNA-Seq data of placental tissues from the preterm pregnancies in GSE73685. Genes with expression levels lower than 0.1 in all tissues in FANTOM5 are defined as unexpressed genes. Placenta-enriched, unexpressed, top500, and bottom500 genes are shown in S1–-S3 Tables. The pTSS region is denoted by grey dashed lines. The areas with light colors along the mean lines represent standard error of mean (SEM). PTB,  preterm birth; TSS,  transcriptional start site; cfDNA,  cell-free DNA.

We first compared the read coverage at the pTSS (primary transcription start site, defined as the region ranging from −1 to + 1 kb around the transcriptional start site) between the 500 most highly and 500 least expressed genes in the preterm placenta. The 500 most highly expressed genes showed reduced depth at the pTSS regions compared to the 500 least expressed genes (Fig 2A and 2B; *P*-value =  1.9e−06, Wilcoxon Signed-Rank test). Additionally, the housekeeping genes with highly expressed levels exhibited lower read depth, whereas the unexpressed genes exhibited higher read depth (Fig 2C and 2D; *P*-value =  1.9e−06, Wilcoxon Signed-Rank test). Similar patterns were observed in maternal whole blood data (S3 Fig). To ensure the robustness of our findings, we extended our analysis by incorporating additional high/low expression data from four other datasets representing diverse ethnic backgrounds, including samples from South Korea (GSE148402), China (GSE174415), Canada (GSE59491 and GSE46510), and the USA (GSE73685). These results are consistent with those in Figs 2B, 2D, and S4, supporting the reliability of our methodology. Therefore, we confirmed that the coverage of plasma cfDNA at the pTSS regions closely correlates with the expression profiles of its original tissues, suggesting that promoter profiling could reflect the expression status of its original tissues. Next, we focused on the cfDNA profiles of placenta-enriched genes, which were closely related to placental functions. The results revealed that placenta-enriched genes were characterized by reduced coverage at the pTSS regions in PTB pregnancies compared to full-term pregnancies (Fig 2E; *P*-value =  7.386e−11, Wilcoxon rank-sum test), implying that there may be broad differences in the promoter profiling of these two groups.

### Promoter profiling of cfDNA revealed distinct patterns between PTB and controls

**Fig 3:**
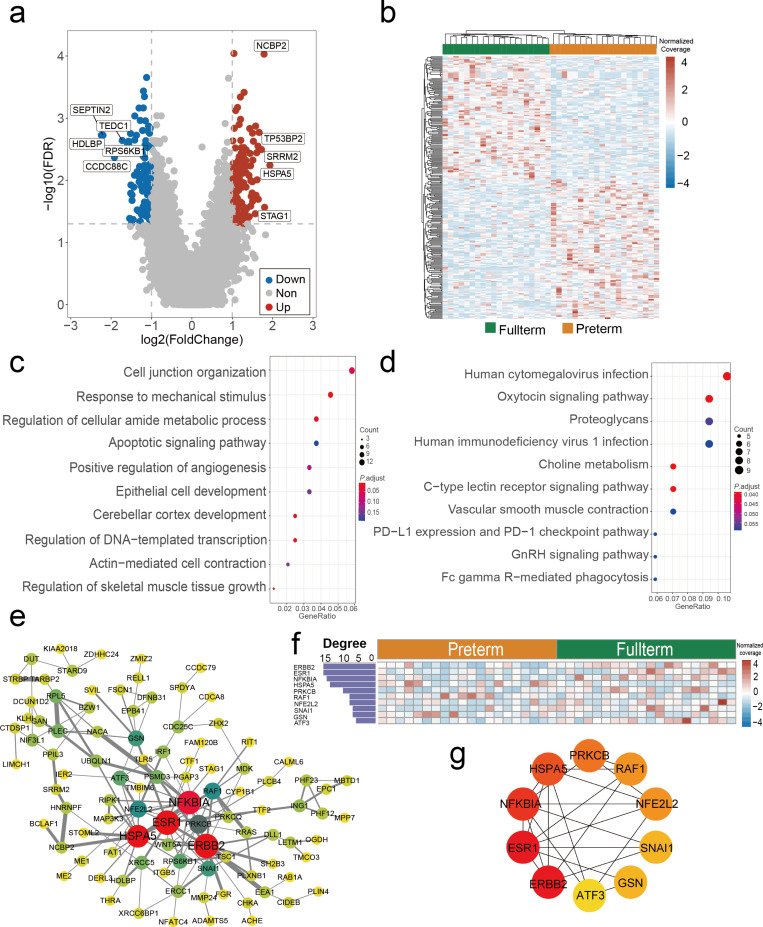
Differences in pTSS profiles between pre- and full-term pregnancies. (a) Volcano plots showing genes with differential read coverages within the pTSS regions (spanning from −1 to +1 kb around TSS) between 20 preterm birth (PTB) and 20 full-term pregnancies. A total of 277 transcripts with differential read coverages within pTSS regions were identified (|log_2_ fold change | ≥  1 and false discovery rate [FDR] ≤  0.05). The red, blue, and grey dots indicate transcripts with increased, decreased, and non-differential coverage, respectively. The x- and y-axes represent the log fold change and *P*-value, calculated by the two-sided Wilcoxon rank-sum test, respectively (*n* = 40, including 20 preterm and 20 full-term pregnancies). The raw *P-*value was adjusted to the false discovery rate (FDR) using the Benjamini-Hochberg procedure. The top five up-regulated and down-regulated genes are marked in the volcano plot. **(b)** Heatmap showing the *z*-scores of genes with differential read coverages at pTSS, generated by the pheatmap package (ver. 1.0.2) using the complete-linkage clustering algorithm. **(c)** Gene Ontology enrichment analysis of transcripts with differential coverage between PTB and full-term groups using Metascape (ver. 20220101). **(d)** KEGG pathway enrichment analysis of transcripts with differential coverage between PTB and full-term groups using clusterProfiler (ver. 3.18.1). **(e)** Gene correlation network for transcripts with differential coverage between PTB and full-term groups, with gene correlations evaluated using the String database (ver. 11.5) and network visualization by Cytoscape (ver. 3.8). **(f)** Degrees and heatmap of hub gene interconnections within the correlation network. The degree represents the importance of the genes in the network, evaluated by cytoHubba (ver. 0.1). **(g)** Correlation network of hub genes.

We then investigated whether cfDNA promoter profiling of pre- and full-term pregnancies demonstrated any deviations in patterns. By comparing their cfDNA promoter profiling, we identified 277 genes with differential coverages at the pTSS (Fig 3A; | Log_2_ fold change | >  1 and FDR <  0.05). These genes included 146 genes with increased coverage and 131 genes with decreased coverage (S5 Table). Next, we used PCA on these genes and found that samples from the same group had similar promoter profiling (S5 Fig). More importantly, unsupervised clustering analysis produced a heatmap that revealed distinct differences in promoter coverage for pre- and full-term pregnancies (Fig 3B).

GO and KEGG enrichment analyses were used to annotate the functions of the genes with differential coverages at pTSS. The results of GO enrichment showed that the terms associated with cell junction organization, response to mechanical stimulus, apoptosis, and development were closely related to embryonic development and premature delivery (Fig 3C). For instance, previous studies have shown that apoptosis of fetal membranes could plausibly contribute to the risk of PTB [36]. Additionally, KEGG enrichment analysis revealed that a large proportion of the enriched pathways were closely associated with embryonic development and PTB (Fig 3D).

Finally, we sought to find the potential key genes associated with PTB using a gene correlation network (Fig 3E). The analysis of gene functional connections allowed us to evaluate the degree of gene influence and importance, which may help identify essential genes in the occurrence and progression of PTB. According to gene degree, our evaluations identified the top 10 hub genes with the maximum degree (Fig 3F). These genes included *ERBB2*, *ESR1*, *NFKBIA*, *HSPA5*, *PRKCB*, *RAF1*, *NFE2LE*, *SNAI1*, *GSN*, and *ATF3* (Fig 3F)*.* The correlation network showed the close relationship among the hub genes (Fig 3G). Literature retrieval revealed that the 10 hub genes were associated with PTB, embryonic development, and pregnancy (S8 Table). For example, the *ESR1* gene polymorphism is associated with premature delivery, with its DNA methylation patterns also showing distinct differences between pre- and full-term pregnancies [37,38]. Similarly, *NFKBIA* degradation could activate NF-κB, resulting in the production of proinflammatory IL-6, and inflammation is closely associated with PTB [39]. In particular, the expression of *ATF3* is significantly decreased in preterm placentas, and *ATF3* is the regulator of soluble fms-like tyrosine kinase 1 (sFlt-1) and soluble Endoglin (sEng), which are important markers of premature delivery and preeclampsia [40].

### Promoter profiling of plasma cfDNA can predict PTB

**Fig 4:**
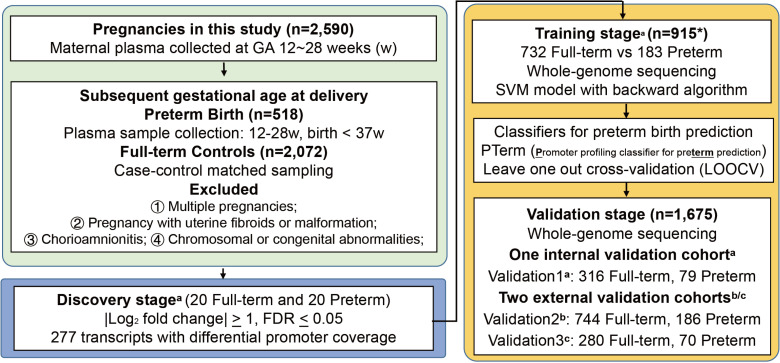
Pipeline for preterm birth classifier construction. In this study, 2,590 plasma cfDNA samples (518 preterm birth and 2,072 full-term pregnancies) were collected from three independent hospitals, including Jiangmen maternal and child healthcare hospital (JM)^a^, Foshan maternal and child healthcare hospital (FS)^b^, and Nanfang Hospital of Southern Medical University (NFY)^c^. These samples were collected between 12 and 28 weeks of gestation. According to their subsequent delivery time, the pregnant women were categorized into preterm or full-term groups. The whole-genome sequencing data was then used to develop classifiers for predicting PTB via a three-step process: discovery, training, and validation. In the discovery stage, we identified 277 transcripts with differential coverage at pTSS regions (spanning from −1 to +1 kb around TSS) between the two groups. * The 20 preterm and 20 full-term pregnancies derived from JM used in the discovery stage were also included in the 915 samples of the training stage. In the training stage, we applied non-linear support vector machine (SVM), logistic regression (LR), random forest (RF), and XGBoost (XGB) models, independently augmented with backward and Lasso feature selection algorithms to develop a set of predictive classifiers. The predictive classifier, denoted by PTerm, achieved the largest area under the curve (AUC) was identified and its performance was further validated in the three validation cohorts, including one internal cohort (validation1, JM: *n* =  395) and two external cohorts (validation2 derived from FS: *n* =  930; Validation3 derived from NFY: *n* =  350). Additional details about participant definitions and classifier construction are provided in the Methods section and S1 Text. PTB, preterm birth; GA, gestational age.

**Fig 5:**
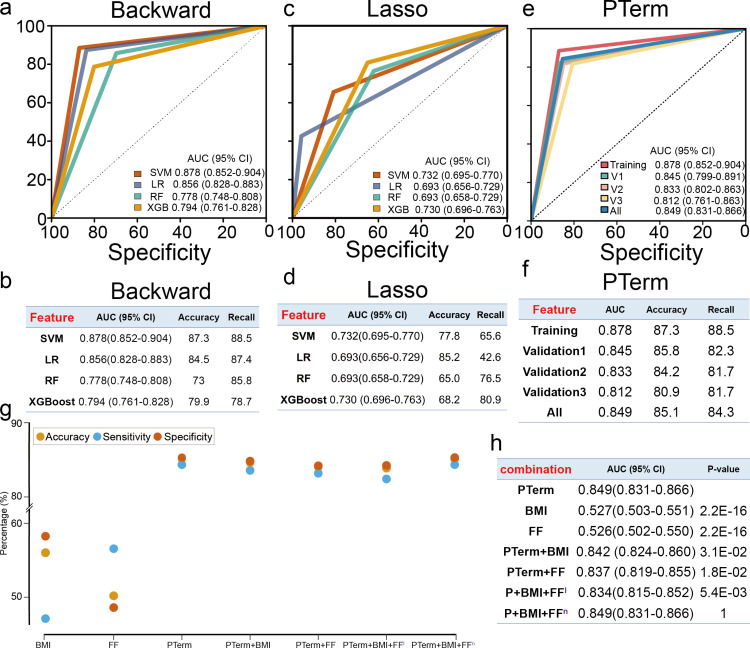
Performance of the classifiers in predicting preterm birth. (a) Receiver operating characteristic (ROC) curves for each of the predictive classifiers using the backward feature selection algorithm. (b) Performance of each of the predictive classifiers using the backward feature selection algorithm. (c) ROC curves for the predictive classifiers using the Lasso feature selection algorithm. (d) Performance of the classifiers using the Lasso feature selection algorithm. (e) ROC curves of the optimal classifier, PTerm. (f) Performance of PTerm across each cohort. (g) Performance of different combinations. (h) Area under the curves (AUCs) of different combinations. In this study, 2,590 plasma cfDNA samples (518 preterm births and 2,072 full-term births) were collected from three independent hospitals. In the training stage, the predictive classifiers were trained using support vector machine (SVM), logistic regression (LR), random forest (RF), and XGBoost (XGB) with backward and Lasso feature selection algorithms (*n* =  915). Leave-one-out cross-validation (LOOCV) was used to evaluate the performance of the selected classifiers. In the validation stage, the performance of the classifier was evaluated in three cohorts, including the internal cohort (validation1, *n* =  395) and two external cohorts (validation2, *n* =  930; validation3, *n* =  350). The significant differences in ROC curves were compared using the two-sided DeLong’s test. FF, fetal fraction; BMI, body mass index before pregnancy; P+BMI+FF^l^, the combination of PTerm, BMI, and fetal fraction using the linear kernel of SVM model; P+BMI+FF^n^, the combination of PTerm, BMI, and fetal fraction using the non-linear kernel (RBF kernel) of SVM model.

To further validate the potential of cfDNA promoter profiling in predicting PTB, we established a large-scale, multi-center, case-control study that included 2,590 pregnant women, consisting of 518 preterm and 2,072 full-term pregnancies, from three independent hospitals (Fig 4). According to the rank of gene importance and gene correlation coefficient, highly related genes were filtered, and 228 genes were retained (S5 Table). Our training stage focused on the 228 genes with differential coverage at the pTSS identified in the discovery stage. We then used four predictive models (SVM, LR, RF, and XGB) and two feature selection algorithms (backward and lasso algorithms) to develop the optimal predictive classifier. We found that the AUC of the optimal classifiers for each model based on the backward feature selection algorithm was significantly higher than those of classifiers with the lasso algorithm (Fig 5A–5D and S9 Table; all *P*-values <  0.05, DeLong’s test). More importantly, we found that a classifier that relied on the SVM model and backward algorithm, named PTerm, performed well as the best predictor of PTB (AUC =  0.878 [0.852–0.904], accuracy =  87.3%, and recall =  88.5%; S10 Table). PTerm exhibited the largest AUC value after LOOCV (0.878 [0.852–0.904]), and its AUC value was higher than those of the optimal classifiers produced using the LR, RF, and XGB models (Fig 5A and 5B and S11 Table).

In PTerm, the classifier contains 83 genes with a linear SVM model (S7 Table). In addition, 4 of the 10 hub genes (*ERBB2*, *NFKBIA*, *RAF1*, and *GSN*) were retained in the classifier. Then, we evaluated the performance of PTerm across three validation cohorts, including one internal and two external validation cohorts. Consistent with the results of the training cohort, PTerm exhibited solid predictive capacity in all three cohorts. The AUC for the internal validation cohort was 0.845 (0.799–0.891), and the AUCs for external validation 2 and validation 3 cohorts were 0.833 (0.802–0.863) and 0.812 (0.761–0.863), respectively (Fig 5E and 5F). In addition, PTerm produced an AUC of 0.849 (0.831–0.866) across all cohorts when discriminating between pre- and full-term pregnancies, with a recall of 84.4% and an accuracy of 85.3% (Fig 5E and 5F and S10 Table).

PTB is a diverse etiology with some causes being iatrogenic and others being wholly spontaneous. Spontaneous labor with intact membranes and PPROM are the main types of PTB. In our study, we found that spontaneous labor with intact membranes accounted for 53.1% of spontaneous preterm pregnancies, with a prediction accuracy of 0.855 (S6 Fig). Meanwhile, PPROM accounted for 46.9% of spontaneous preterm pregnancies, with a prediction accuracy of 0.823 (S6 Fig). Additionally, the pathogenic factors of premature delivery in different birth weeks may be different. For our prediction, the accuracy for preterm pregnancies with birth before 35 weeks reached 0.866 (S12 Table), demonstrating the potential application value of our method.

### PTerm combined with clinical features

Previous studies have reported that certain clinical features, such as fetal fraction (FF) and BMI before pregnancy, could be used to predict PTB. In our data, we found that the AUCs of BMI (0.527 [0.503–0.551]) and FF (0.526 [0.502–0.550]) were significantly lower than that of PTerm (Fig 5G and 5H; all *P*-values <  0.05, DeLong’s test). To improve the performance of our classifier, we combined the features of BMI and FF with PTerm. These clinical features were incorporated as variables alongside the 83 original variables in PTerm to construct the classifiers. The AUCs of the combined classifiers (PTerm + BMI, PTerm + FF, and PTerm + BMI + FF) were 0.842 (0.824–0.860), 0.837 (0.819–0.855), and 0.834 (0.815–0.852), which were also significantly lower than that of PTerm alone (Fig 5G and 5H; all *P*-value <  0.05, DeLong’s test). Previous studies have shown that the clinical features (FF and BMI) might exhibit a non-linear association with premature delivery. Therefore, we applied the non-linear kernel of the SVM model to integrate PTerm with BMI and FF (Integration of clinical features in S1 Text). We found that the AUC of the combined classifiers was 0.849 (0.831–0.866), which was significantly higher than that of the linear model (0.834 [0.815–0.852]; *P*-value =  0.0054, DeLong’s test) and equal to that of PTerm alone (Fig 5G and 5H). The performance of PTerm when combined with cfDNA and clinical variables (0.833 [0.814–0.851]) was significantly higher than that of clinical variables alone (0.527 [0.504–0.551]), highlighting the clinical utility of our novel model (*P*-value < 2.2e−16, DeLong’s test).

## Discussion

In this study, we described the application of promoter profiling of plasma cfDNA to predict premature delivery. We found that promoter profiling of cfDNA reflects the expression status of its tissues of origin, and broad changes in promoter profiling were observed between pre- and full-term pregnancies. Given this, we hypothesized that differential read-depth patterns of cfDNA at promoters should supply sufficient information regarding placenta-origin diseases long before any clinical symptoms would appear. Thus, we developed a series of predictive classifiers using data from large-scale multi-center cohorts (*n* = 2,590), which included pregnancies from three independent centers. These classifiers were developed using four machine learning models and two feature selection methods to ensure optimal performance. This development produced a robust predictive classifier, PTerm, which was shown to predict PTB with an overall AUC of 0.849 (0.831–0.866). These findings highlight the potential value of promoter profiling of cfDNA as a non-invasive method for predicting preterm delivery at an early gestational age.

The promoter profiles of cfDNA may reflect gene expression patterns of maternal blood and placental tissues, enabling the identification of biological pathways plausibly linked to underlying physiological changes that take place in early pregnancy. First, gene function enrichment results showed that several pathways were closely related to PTB, such as apoptosis and oxytocin signaling pathways. As an example, premature activation of oxytocin secretion often results in preterm labor, and oxytocin receptor antagonists could inhibit PTB [41]. Additionally, 10 hub genes were related to PTB, including *ESR1*, *NFKBIA*, and *ATF3* [37–40]. More importantly, literature review revealed that several genes in PTerm are associated with preterm-related processes (S13 Table). These signaling and metabolic pathways may provide clinically targetable pathways and biomarkers, and aid in identifying potential therapeutic targets.

Placental dysfunction is a leading cause of premature delivery. Our studies have revealed a close relationship between placental expression profiles and promoter profiles of cfDNA. Analyzing the fetal fraction in our datasets, we observed no significant difference in fetal fraction between pre- and full-term birth pregnancies in early gestation (S14 Table), consistent with previous studies [42,43]. Although the difference in fetal fraction is not significant, investigating the placental contribution to the genes with differential coverages holds profound importance. By comparing promoter profiles, we found 277 genes with differential promoter coverages closely associated with premature delivery. To further explore the sources of cfDNA, more data, such as cfDNA methylation, are needed to elucidate the contributions of different organs. Given the pivotal role of placental dysfunction in premature delivery, identifying its contribution to the genes with differential coverages may uncover placental-related pathogenic factors, thus providing substantial benefits for the treatment and management of premature delivery.

Recent studies have made pioneering attempts to use maternal blood omics data (cfDNA, cfRNA, and metabolites) to predict future complications in pregnancy, such as PTB and preeclampsia [3–5,35,44,45]. Biomarker studies require large sample sizes, high-throughput screening, and independent cohort validation. To date, few studies have recruited more than 2,500 samples with high-throughput screening and performed validation in multiple independent cohorts. In this study, we collected 2,590 whole-genome sequencing datasets of plasma cfDNA derived from 518 preterm and 2,072 full-term pregnancies from three independent hospitals to train and validate the classifiers for predicting PTB. Additionally, useful biomarkers for disease prediction need to be stable, non-invasive, and low-cost. cfDNA meets these needs, and its detection via NIPT has been widely used for fetal trisomy detection worldwide. In 2018, 10 million NIPT tests were performed in over 60 countries [46]. Since PTerm can utilize current NIPT data without changing its procedure or adding detection costs, it can be easily adapted for preclinical tests.

Nevertheless, our study has several limitations. Prior studies indicate that ethnic backgrounds influence risk factors for PTB. To assess the generalizability of PTerm, additional samples from ethnically diverse populations and various countries are required. Moreover, variability in the timing of sample collection during the second trimester may affect PTerm’s performance. Future studies should investigate the impact of sample timing on PTerm’s accuracy, given that cfDNA profiles exhibit dynamic changes across gestational stages and pathological conditions. Although PCA analysis demonstrated that promoter profiles of cfDNA can effectively differentiate PTBs from healthy pregnancies in the discovery cohort, PTB remain a multifaceted and complex complication. Accurate prediction requires the application of advanced classification and prediction algorithms, including SVM, LR, and RF, to capture complex patterns and enhance predictive accuracy. Finally, the absence of histological analysis of placenta pathology limits insights into the underlying mechanisms of PTB. Incorporating placental histopathological data could not only refine predictive models but also facilitate the identification of novel therapeutic targets. To support the clinical translation of PTerm, prospective studies are essential to validate its utility and further elucidate the molecular mechanisms underlying PTB, thereby guiding the development of targeted interventions.

## Conclusions

In summary, our data suggest that the promoter-profiling-based classifier (PTerm) could provide valuable PTB predictions in early pregnancy. Our method is also easily applicable to routine NIPT data and does not require any additional tests or increase detection costs, making it feasible in clinical practice. Given this, we believe that our method serves as a critical stepping stone toward developing a non-invasive diagnostic for the early prediction of pregnancy complications. Currently, PTerm can distinguishing PTB pregnancies from full-term pregnancies with high accuracy. Moving forward, leveraging additional data on promoter profiles across different gestational ages could facilitate developing a model for accurately predicting delivery time.

## Supporting information

S1 Text Supplement to method details. (DOCX)

S1 Fig Flowchart of classifier construction. (DOCX)

S2 Fig Flowchart of exclusion of preterm pregnancies. (DOCX)

S3 Fig cfDNA profiles at promoter region reflect nucleosome positioning of blood cells in preterm birth pregnancies. (DOCX)

S4 Fig cfDNA profiles at promoter region reflect nucleosome positioning of placenta and whole blood cells in more preterm birth studies S3 Fig. (DOCX)

S5 Fig Principal component analysis (PCA) of the genes with differential coverages. (DOCX)

S6 Fig The ratios and predictive accuracy of spontaneous labor with intact membranes and preterm premature rupture of the membranes (PPROM). (DOCX)

S1 Table 500 highest/lowest expressed genes in placenta. (DOCX)

S2 Table Placenta- and whole blood-enriched genes. (DOCX)

S3 Table Housekeeping and unexpressed genes. (XLXS)

S4 Table Clinical characteristics of pregnancies in the discovery cohort. (DOCX)

S5 Table Genes with differential read coverages at the pTSS. (DOCX)

S6 Table Correlation coefficients of the filtered genes. (DOCX)

S7 Table The genes in PTerm modelS8 Table. Functional annotation of the hub genes by retrieving literature. (DOCX)

S9 Table Performance of the optimal classifier for each model with backward and lasso algorithm. (DOCX)

S10 Table Performance of the classifiers. (DOCX)

S11 Table Comparison of the predictive efficacy of PTerm with and optimal classifiers from other models. (DOCX)

S12 Table The accuracy of predicting premature delivery in different birth weeks. (DOCX)

S13 Table Functional annotation of genes in PTerm by retrieving literatures. (DOCX)

S14 Table The comparison of fetal fraction between pre- and full-term pregnancies. (DOCX)

## Abbreviations

AUC: area under the curve
BMI: body mass index
cfDNA: cell-free DNA
cffDNA: cell-free fetal DNA
cfRNA: cell-free RNA
CV: cross-validation
FDR: false discovery rate
FF: fetal fraction
FS: Foshan maternal and child healthcare hospital
GA: gestational age
GO: gene ontology
IQR: interquartile range
JM: Jiangmen maternal and child healthcare hospital
LOOCV: leave-one-out cross-validation
LR: logistic regression
NFY: Nanfang hospital of southern medical university
NIPT: non-invasive prenatal testing
PCA: principal component analysis
PTB: preterm birth
PPROM: premature rupture of the membranes
PTerm: promoter profiling classifier for preterm prediction
RF: random forest
ROC: receiver operating characteristic
sFlt-1: soluble fms-like tyrosine kinase 1
sEng: soluble Endoglin
sPTB: spontaneous preterm birth
SVM: support vector machine
TSS: transcriptional start site
XGB: XGBoost
