# Supplementary material for: Genome-wide nucleosome footprints of plasma cfDNA predict preterm birth: A case-control study
Source: PLoS Med. 2025 Apr 15;22(4):e1004571. doi: 10.1371/journal.pmed.1004571 (PMC11999135; doi:10.1371/journal.pmed.1004571)
Supplement: S1 Text — (DOCX) [file pmed.1004571.s001.docx]

Supporting Information

**Genome-wide Nucleosome Footprints of Plasma cfDNA Predict Preterm Birth**

*Zhiwei Guo*^†^, *Ke Wang*^†^, *Xiang Huang*^†^, *Kun Li*, *Guojun Ouyang*, *Xu Yang*, *Jiayu Tan*, *Haihong Shi*, *Liangping Luo*, *Min Zhang*, *Bowei Han*, *Xiangming Zhai*, *Jinhai Deng*, *Richard Beatson*, *Yingsong Wu*^*^, *Fang Yang*^*^, *Xuexi Yang*^*^, *Jia Tang*^*^

**Supplemental methods**

1. **Sample collection and cfDNA isolation**

Maternal whole blood was collected using Streck cell-free DNA blood collection tubes (BCT; Streck, USA) and was then centrifuged at 1,600g and 25°C for 15 minutes before plasma collection. This plasma was then centrifuged a second time at 2,500g and 25°C for 10 minutes. After the second spin, the plasma was removed from the pellet that formed at the bottom of the tube, distributed into 4 mL barcoded plasma aliquots and immediately stored frozen at −80°C until DNA extraction. According to the manufacturer’s instructions, cfDNA was extracted from plasma samples using the QIAamp DNA blood mini kit (Qiagen, Germany). Qubit fluorometer (ThermoFisher Scientific, USA) and Agilent 2100 bioanalyzer (Agilent Technologies, USA) were used to measure DNA concentration and integration. The DNA was eluted in 50 µL AE buffer and stored at −20°C. Participants were instructed to stop using aspirin/hairpin within 72 hours before whole blood collection.

1. **Whole-genome Sequencing of plasma cfDNA**

Plasma cfDNA libraries were prepared following the Illumina TruSeq library preparation protocol (Illumina, USA) and AMPure XP magnetic bead clean-up (Beckman Coulter, USA) on a Caliper Zephyr liquid handler (PerkinElmer, USA). The TruSeq indexes were then incorporated into the libraries before each was quantified on the Caliper LabChip GX (PerkinElmer, USA) and normalized to the same concentration These libraries were sequenced using the NextSeq platform (Illumina, USA). For each sample, approximately 4.8 million reads of 36-bp were generated.

1. **Statistical power analysis in discovery stage**

To ensure that the difference between 20 preterm cases and 20 full-term controls deemed to be clinically meaningful, the normalized data of the candidate TSSs with the lowest fold change was subjected to statistical power analysis.^1^ The power was calculated by 1-β, in which β was measured as follows:

$$Z_{\beta}=\frac{\delta}{\sigma\sqrt{\frac{1}{n^{Preterm}}+\frac{1}{n^{controls}}}}-Z_{\alpha/2}$$

In this equation,$\alpha$= 0.05, $Z_{\alpha/2}$= 1.96; $n^{Preterm}$= 20, $n^{controls}$= 20, $\delta$=$|\bar{n}^{Preterm}-\bar{n}^{controls}|$= 18.92733, $\sigma=(\sigma^{Preterm}+\sigma^{controls})/2$= 21.51462. When the result of power was higher than 80%, the samples size was adequate to detect differences of clinical importance.

**4.** **Integration of clinical features**

To integrate PTerm with clinical features, including FF and BMI, both linear and non-linear kernel of SVM model were applied. To determine the parameters for the kernel parameter γ and regularization parameter C in a non-linear kernel (RBF kernel), the grid search method was employed within the grid search region of –5≤log_2_ γ≤5 and –5≤log_2_ C≤ 5. Finally, γ=1 and C=4 was selected as the parameters of RBF kernel.

**Reference:**

1. Lachin JM. Introduction to sample size determination and power analysis for clinical trials. *Control Clin Trials*. 1981; 2: 93-113.
