# Supplementary material for: Genome-wide nucleosome footprints of plasma cfDNA predict preterm birth: A case-control study
Source: PLoS Med. 2025 Apr 15;22(4):e1004571. doi: 10.1371/journal.pmed.1004571 (PMC11999135; doi:10.1371/journal.pmed.1004571)
Supplement: S1 Fig — (DOCX) [file pmed.1004571.s002.docx]

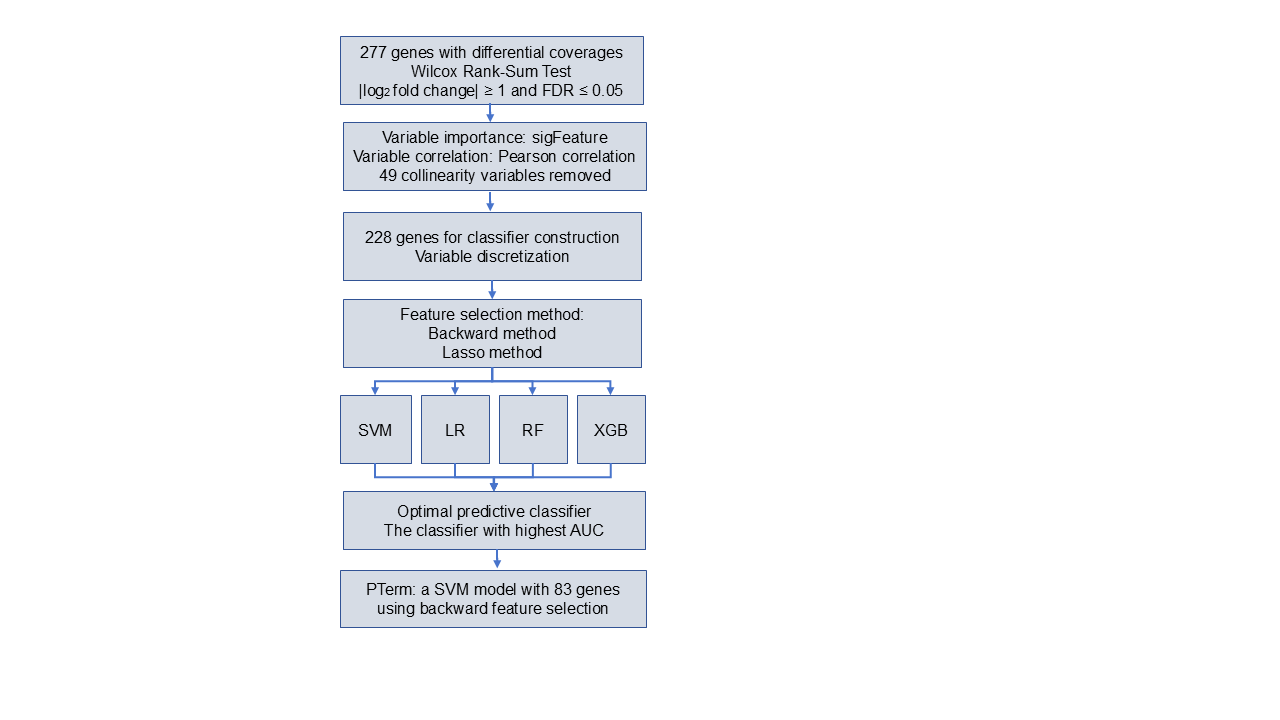


**S1 Fig. Flowchart of classifier construction.** The Wilcoxon rank-sum test was employed to identify genes with differential coverage, characterized by a |log_2_ fold change| ≥ 1 and FDR ≤ 0.05. Pearson correlation coefficients were calculated for all pairs of genes exhibiting differential coverages. Genes exhibiting with high collinearity were assessed (|r| > 0.5), and those with higher importance were retained. This process excluded 49 genes, retaining a total of 228 genes. Three models were developed using both backward and lasso feature selection methods. Among all classifiers, the classifier achieving the highest AUC after cross-validation were selected. SVM= support vector machine. LR=logistic regression. RF= random forest. XGB= XGBoost.
