# Supplementary material for: Genome-wide nucleosome footprints of plasma cfDNA predict preterm birth: A case-control study
Source: PLoS Med. 2025 Apr 15;22(4):e1004571. doi: 10.1371/journal.pmed.1004571 (PMC11999135; doi:10.1371/journal.pmed.1004571)
Supplement: S2 Fig — (DOCX) [file pmed.1004571.s003.docx]

**
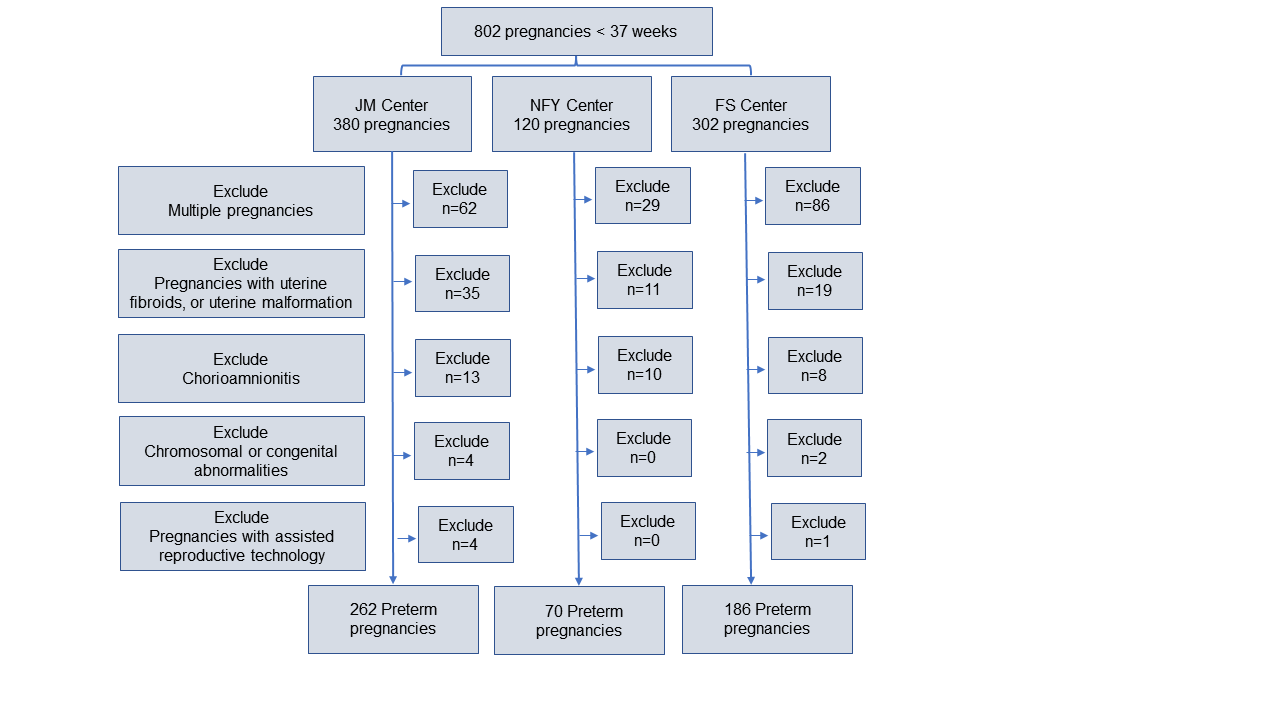
**

**S2 Fig. Flowchart of exclusion of preterm pregnancies.** Pregnancies were excluded in the following order: multiple pregnancies; pregnancies with uterine fibroids or uterine malformation; chorioamnionitis; Chromosomal or congenital abnormalities; pregnancies with assisted reproductive technology.
