# Supplementary material for: Genome-wide nucleosome footprints of plasma cfDNA predict preterm birth: A case-control study
Source: PLoS Med. 2025 Apr 15;22(4):e1004571. doi: 10.1371/journal.pmed.1004571 (PMC11999135; doi:10.1371/journal.pmed.1004571)
Supplement: S3 Fig — (DOCX) [file pmed.1004571.s004.docx]

**
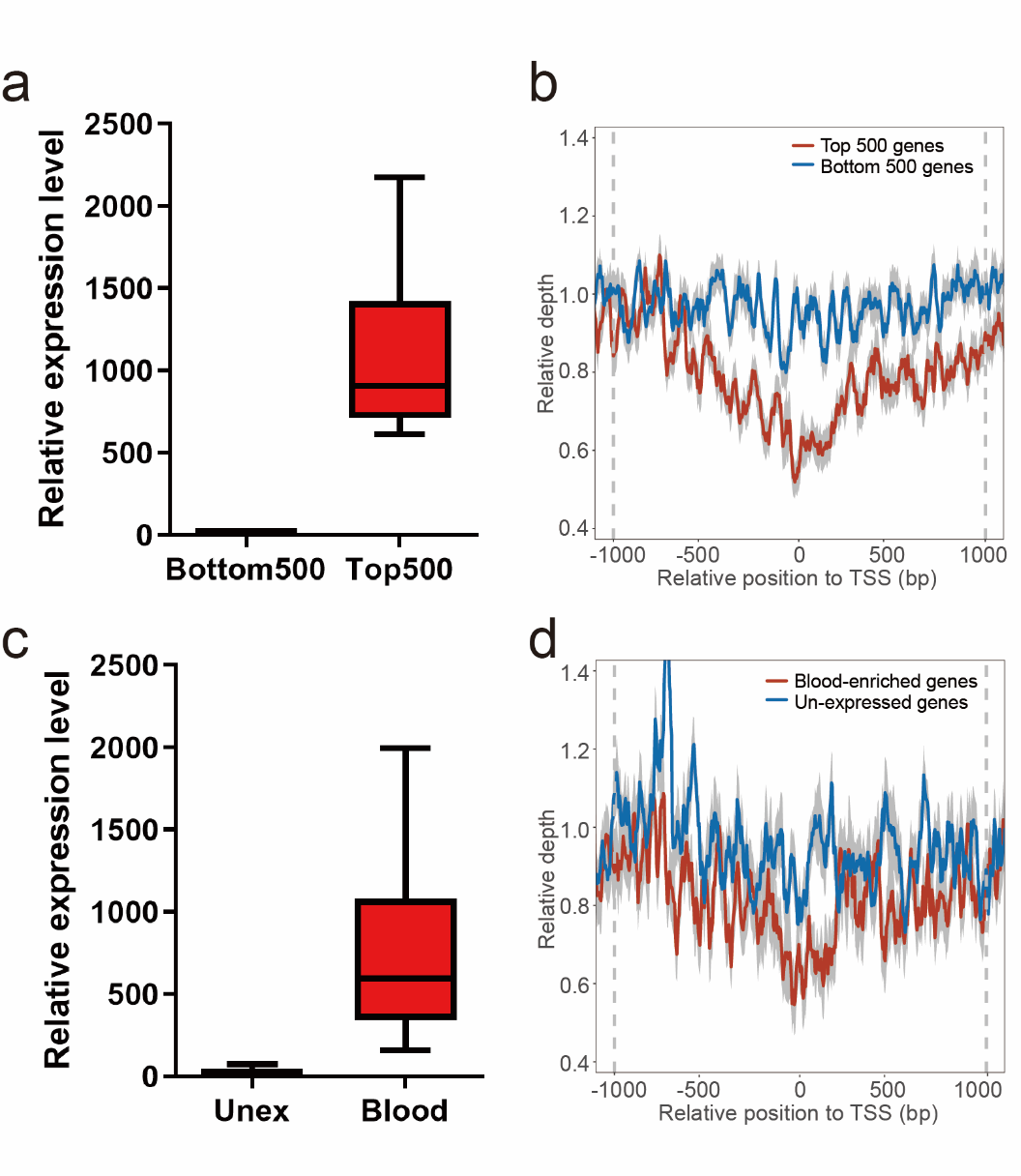
**

**S3 Fig. cfDNA profiles at promoter regions reflect nucleosome positioning of blood cells in pregnancies. a** Average expression levels of the 500 most highly expressed genes (Top500, red) and the 500 least expressed genes (Bottom500, blue) in the blood of preterm birth pregnancies. **b** Read depth of whole-genome sequencing at the pTSS region (-1KB to 1KB around the TSS) of the 500 most highly expressed genes (Top500, red line) and the 500 least expressed genes (Bottom500, blue line). **c** Average expression levels of the blood-enriched genes (blood, red) and the unexpressed genes (Unex, blue) in the blood of preterm birth pregnancies. **d** Read depth of whole-genome sequencing at pTSS region (-1KB to 1KB around TSS) of the blood-enriched genes (red line) was lower than that of unexpressed genes (blue line). Top500 and bottom500 genes are based on the RNA-Seq data of the placental tissues from the preterm pregnancies in GSE73685. The list of blood-enriched genes, unexpressed genes, top500 and bottom500 genes were shown in Supplemental Table S1-S3. pTSS region (-1KB to 1KB around the TSS) was marked between grey dash lines. The areas with light colors along with the mean lines represent standard error of mean (SEM). PTB, preterm birth; TSS, transcriptional start site; cfDNA, cell-free DNA.
