# Supplementary material for: Genome-wide nucleosome footprints of plasma cfDNA predict preterm birth: A case-control study
Source: PLoS Med. 2025 Apr 15;22(4):e1004571. doi: 10.1371/journal.pmed.1004571 (PMC11999135; doi:10.1371/journal.pmed.1004571)
Supplement: S4 Fig — (DOCX) [file pmed.1004571.s005.docx]

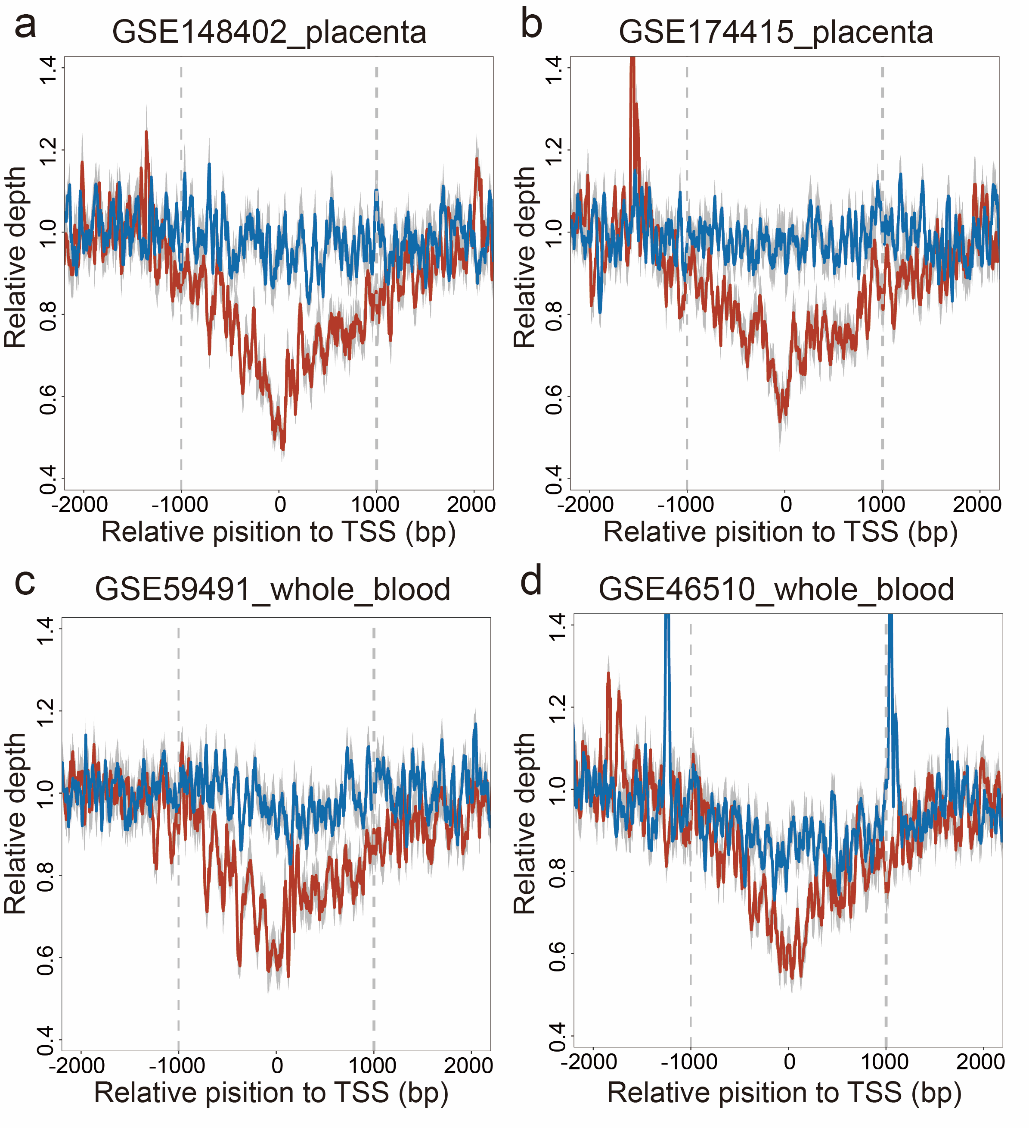


**S4 Fig. cfDNA profiles at promoter regions reflect nucleosome positioning of placenta and whole blood cells in preterm pregnancies.** Read depth of the whole-genome sequencing at the pTSS region (-1KB to 1KB around the TSS) of the 500 highest expressed genes (Top500, red line) and the 500 lowest expressed genes (Bottom500, blue line). **a** The mRNA expression profiles of placenta derived from GSE148402; **b** The mRNA expression profiles of placenta from GSE174415; **c** The mRNA expression profiles of whole blood cells from GSE59491; **d** The mRNA expression profiles of whole blood cells from GSE46510. The areas with light colors along with the mean lines represent the standard error of the mean (SEM). TSS, transcriptional start site; cfDNA, cell-free DNA. GSE148402 from South Korea; GSE174415 from China; GSE59491 and GSE46510 from Canada; GSE73685 used in Figure 2, from USA.
