# Supplementary material for: Genome-wide nucleosome footprints of plasma cfDNA predict preterm birth: A case-control study
Source: PLoS Med. 2025 Apr 15;22(4):e1004571. doi: 10.1371/journal.pmed.1004571 (PMC11999135; doi:10.1371/journal.pmed.1004571)
Supplement: S5 Fig — (DOCX) [file pmed.1004571.s006.docx]

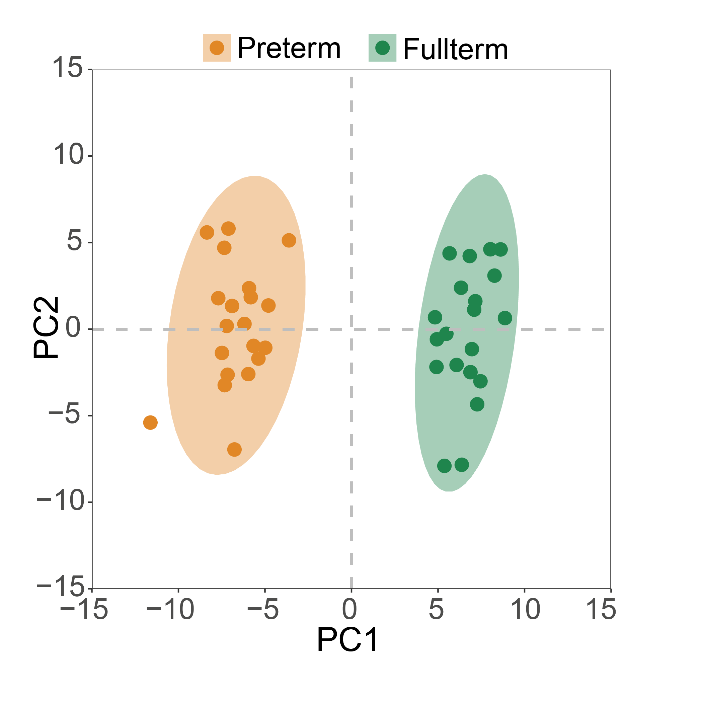


**S5 Fig. Principal Component Analysis (PCA) of the genes with differential coverages.** PC1 and PC2 represent the first and second component of PCA analysis, respectively.
