# Supplementary material for: Genome-wide nucleosome footprints of plasma cfDNA predict preterm birth: A case-control study
Source: PLoS Med. 2025 Apr 15;22(4):e1004571. doi: 10.1371/journal.pmed.1004571 (PMC11999135; doi:10.1371/journal.pmed.1004571)
Supplement: S6 Fig — (DOCX) [file pmed.1004571.s007.docx]

**
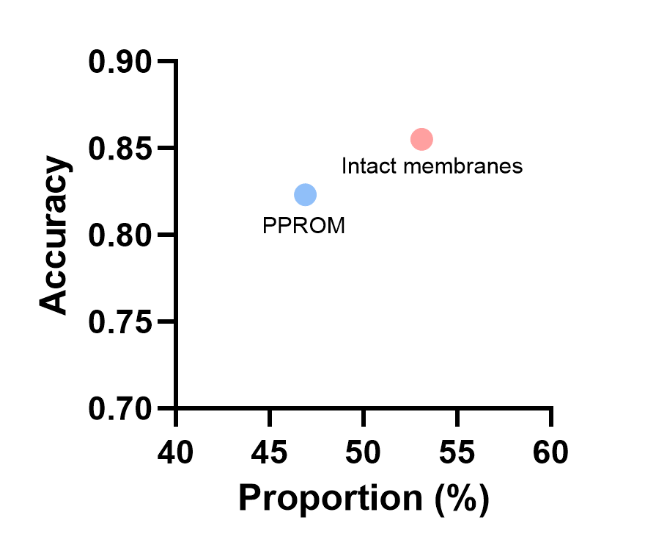
**

**S6 Fig. The proportion and predictive accuracy of spontaneous labor with intact membranes and PPROM.** Accuracy = the accuracy of PTerm for two kinds of pregnancies. Proportion = the proportion of two kinds of pregnancies in premature delivery. Intact membranes = spontaneous labor with intact membranes. PPROM= preterm premature rupture of the membranes.
