# Supplementary material for: Genome-wide nucleosome footprints of plasma cfDNA predict preterm birth: A case-control study
Source: PLoS Med. 2025 Apr 15;22(4):e1004571. doi: 10.1371/journal.pmed.1004571 (PMC11999135; doi:10.1371/journal.pmed.1004571)
Supplement: S1 Table — (DOCX) [file pmed.1004571.s008.docx]

**S1 Table. 500 highest/lowest expressed genes in placenta of preterm pregnancies**

| **Number** | **Top500** | **Bottom500** |
| --- | --- | --- |
| 1 | GAPDH | MS4A13 |
| 2 | FN1 | CAPZA3 |
| 3 | MYL6 | QRFPR |
| 4 | UBC | PPP1R42 |
| 5 | RPL30 | C14orf39 |
| 6 | RPS16 | LRRIQ3 |
| 7 | IGFBP1 | OR5T1 |
| 8 | LGALS1 | GUCA1C |
| 9 | EEF1A1 | LRRC66 |
| 10 | RPS8 | OR6C70 |
| 11 | RPLP2 | OR5L2 |
| 12 | ACTB | IL2 |
| 13 | RPLP1 | OR5B2 |
| 14 | RPS25 | OR5T3 |
| 15 | RPL27 | UBE2U |
| 16 | HBB | SLC7A13 |
| 17 | SERPINE1 | MGAT4D |
| 18 | TMSB10 | SULT1E1 |
| 19 | CD63 | CCDC178 |
| 20 | TIMP3 | SPINK14 |
| 21 | SERPING1 | WDR17 |
| 22 | ZNF43 | RNF133 |
| 23 | HSP90B1 | PTH |
| 24 | IGFBP3 | TDGF1 |
| 25 | RPS12 | ADAD1 |
| 26 | FTL | LIPF |
| 27 | RPL13A | C4orf22 |
| 28 | TGM2 | SYCP1 |
| 29 | ACTG1 | NPY5R |
| 30 | CD68 | TAS2R8 |
| 31 | RPL9 | DEFB128 |
| 32 | DCN | ANKRD7 |
| 33 | RHOA | DEFB127 |
| 34 | RPL17 | MROH9 |
| 35 | RNASEK | OR10X1 |
| 36 | RPS3 | DEFB114 |
| 37 | MT2A | OR10R2 |
| 38 | RPL11 | CCDC172 |
| 39 | RPS14 | CCDC175 |
| 40 | RPS20 | OR6C68 |
| 41 | PRG2 | EYS |
| 42 | RPL6 | LIPI |
| 43 | RPS13 | OR8K5 |
| 44 | RPL38 | LMOD3 |
| 45 | RPS6 | KRTAP20-3 |
| 46 | HSPA8 | IFNE |
| 47 | CRYAB | GK2 |
| 48 | LUM | OR6A2 |
| 49 | NACA | TSHB |
| 50 | S100A10 | ADGB |
| 51 | EIF1 | SLCO6A1 |
| 52 | PLCG2 | IAPP |
| 53 | VIM | OTOGL |
| 54 | RPL12 | SPO11 |
| 55 | SPARC | FBXO47 |
| 56 | RNASE1 | RAG2 |
| 57 | RPL35 | CPA6 |
| 58 | TIMP2 | SI |
| 59 | HSPA5 | GABRR3 |
| 60 | ABI3BP | CCDC39 |
| 61 | ARF1 | CYLC2 |
| 62 | RPL18 | GFRAL |
| 63 | TPT1 | RP1 |
| 64 | ANXA2 | IFNA5 |
| 65 | FTH1 | MSTN |
| 66 | CD74 | ADGRG7 |
| 67 | NPC2 | OR8H2 |
| 68 | RPL21 | STATH |
| 69 | KRT8 | TMEM244 |
| 70 | RPS28 | RBM46 |
| 71 | CD248 | TINAG |
| 72 | ITM2B | PRKG2 |
| 73 | RACK1 | ZPBP2 |
| 74 | RBP1 | LRRC19 |
| 75 | NFKBIA | TECRL |
| 76 | ANXA1 | OR8K1 |
| 77 | B2M | HFM1 |
| 78 | FOS | TERB2 |
| 79 | EPAS1 | REG3A |
| 80 | RPL5 | ASIC5 |
| 81 | TUBA1B | STARD6 |
| 82 | SPP1 | ASPA |
| 83 | GNG10 | TAAR1 |
| 84 | SERPINE2 | SLCO1B7 |
| 85 | IGFBP4 | C1orf141 |
| 86 | CTSB | TEX26 |
| 87 | RPS24 | OR5D13 |
| 88 | PFN1 | CLCA1 |
| 89 | IL1R1 | SCP2D1 |
| 90 | RPL26 | TMCO5A |
| 91 | DUSP1 | SCGB1D1 |
| 92 | MFAP5 | TPH1 |
| 93 | LAPTM4A | IL23R |
| 94 | RPL7 | SPINK7 |
| 95 | PFDN5 | BRINP3 |
| 96 | PSAP | TAC1 |
| 97 | APOD | RXFP2 |
| 98 | BHLHE40 | TAS2R9 |
| 99 | MYL9 | C1orf146 |
| 100 | RPSA | TMPRSS11B |
| 101 | CALR | LIPJ |
| 102 | ANXA5 | ZNF726 |
| 103 | RPL3 | OR4S2 |
| 104 | RPL18A | OR8K3 |
| 105 | H3F3A | ADAM7 |
| 106 | PRDX6 | OR13C4 |
| 107 | EIF4G2 | PNLIP |
| 108 | SOD2 | TXNDC8 |
| 109 | ATP5MG | REG1B |
| 110 | PABPC1 | OR4P4 |
| 111 | RPL14 | KIAA1024L |
| 112 | SLC2A1 | F13B |
| 113 | TMBIM6 | DEFB135 |
| 114 | ZFP36L1 | CNTN5 |
| 115 | CD59 | FGL1 |
| 116 | ADAM12 | C1orf87 |
| 117 | SEM1 | OR9A4 |
| 118 | THY1 | MLIP |
| 119 | NUTF2 | CDH12 |
| 120 | IL1R2 | OR5AK2 |
| 121 | ARPC2 | NLRP14 |
| 122 | SLCO2A1 | OR6C2 |
| 123 | SARAF | SLCO1B3 |
| 124 | CANX | TSLP |
| 125 | CXCL8 | KNG1 |
| 126 | TXNIP | NTS |
| 127 | LDHA | OR8J1 |
| 128 | CTSL | MSMB |
| 129 | CCNI | SPATA4 |
| 130 | SEC61G | OR5K4 |
| 131 | TPM3 | OR11G2 |
| 132 | RPL15 | B3GALT1 |
| 133 | FSTL1 | SPAG17 |
| 134 | PAPPA2 | NDST4 |
| 135 | HNRNPC | CDKL4 |
| 136 | NDRG1 | ASZ1 |
| 137 | ZFP36 | DEUP1 |
| 138 | HPGD | SERPINB12 |
| 139 | CCL3 | NR1H4 |
| 140 | RPL10A | TMPRSS11A |
| 141 | GLUL | MAS1 |
| 142 | CD55 | POTEA |
| 143 | HSD3B1 | MEPE |
| 144 | DAD1 | OR4A16 |
| 145 | CITED2 | DYDC1 |
| 146 | TMEM59 | OR5K2 |
| 147 | LCP1 | TERB1 |
| 148 | BASP1 | SPERT |
| 149 | COX6C | ATP6V1G3 |
| 150 | DYNLL1 | CCDC148 |
| 151 | MYL12B | CCDC38 |
| 152 | KRT7 | CCDC158 |
| 153 | HSP90AB1 | PNLIPRP3 |
| 154 | CTSA | C4orf45 |
| 155 | RGCC | RRH |
| 156 | TMBIM1 | KRT28 |
| 157 | JUNB | SLC13A1 |
| 158 | RPL35A | CFAP206 |
| 159 | GABARAP | DEFB112 |
| 160 | YWHAZ | ZSWIM2 |
| 161 | CSDE1 | IL26 |
| 162 | YWHAQ | OR6C65 |
| 163 | NCL | OR51B4 |
| 164 | CGA | CALHM4 |
| 165 | HSP90AA1 | SLC5A8 |
| 166 | CYB5R3 | OR1L1 |
| 167 | MIF | MEIOC |
| 168 | TUBA1A | OR13C8 |
| 169 | BNIP3L | EFCAB1 |
| 170 | ENO1 | SPACA7 |
| 171 | PRDX5 | ANO5 |
| 172 | RAB1A | MSH4 |
| 173 | RPS27A | BEST3 |
| 174 | TNFSF10 | PTTG2 |
| 175 | RPL34 | GLYATL2 |
| 176 | RPS19 | FSTL5 |
| 177 | CST3 | DAOA |
| 178 | CAP1 | RGSL1 |
| 179 | RPL8 | PLEKHG7 |
| 180 | SERINC3 | MORC1 |
| 181 | EEF2 | MYH1 |
| 182 | RPN2 | SULT6B1 |
| 183 | SSR2 | OPRPN |
| 184 | EDF1 | FABP12 |
| 185 | MGST3 | CPB2 |
| 186 | LAPTM5 | EPPIN-WFDC6 |
| 187 | CD44 | UGT2B4 |
| 188 | PAPPA | STK31 |
| 189 | GM2A | LGI1 |
| 190 | MRPL51 | ESM1 |
| 191 | NAMPT | SLC10A5 |
| 192 | RAB7A | ADAM2 |
| 193 | HNRNPA2B1 | CNBD1 |
| 194 | C11orf58 | MIA2 |
| 195 | APP | OR52N4 |
| 196 | GLRX | ADH4 |
| 197 | SLC38A2 | TMPRSS11D |
| 198 | WTAP | ESCO2 |
| 199 | CD164 | OR6C4 |
| 200 | P4HB | C12orf40 |
| 201 | SCAMP2 | OR6K3 |
| 202 | PRL | LIPK |
| 203 | ACTA2 | ANKRD45 |
| 204 | LGMN | PPP1R3A |
| 205 | TXN | ZNF665 |
| 206 | TMED10 | MDH1B |
| 207 | IQGAP1 | ZBBX |
| 208 | TYROBP | BIRC8 |
| 209 | BRI3 | FPGT-TNNI3K |
| 210 | DDX17 | OR4F15 |
| 211 | SELENOT | C4orf17 |
| 212 | OAZ1 | CCDC83 |
| 213 | KDELR2 | DNASE2B |
| 214 | S100A8 | BTG4 |
| 215 | PPIB | MYH8 |
| 216 | CFL1 | SCN7A |
| 217 | HSD11B1 | PPM1K |
| 218 | ATP6V0C | TRDN |
| 219 | HEXB | FSCB |
| 220 | APLP2 | CCR3 |
| 221 | MFAP4 | TMPRSS15 |
| 222 | PPDPF | BCHE |
| 223 | VGLL3 | CAGE1 |
| 224 | CRIM1 | OR8H1 |
| 225 | GJA1 | DEFB110 |
| 226 | DKK1 | SPATA8 |
| 227 | IL6ST | OR13C9 |
| 228 | TFPI2 | FBXO43 |
| 229 | ACTR3 | KRTAP13-2 |
| 230 | SERPINA3 | VIP |
| 231 | SLPI | SCN2A |
| 232 | GPX3 | TEX12 |
| 233 | MMP2 | OR52A1 |
| 234 | HNRNPA1 | OOSP2 |
| 235 | FSTL3 | KRTAP19-6 |
| 236 | TNS4 | SGCZ |
| 237 | HDLBP | RBM11 |
| 238 | RPS2 | STXBP5L |
| 239 | ARF4 | ERICH6 |
| 240 | BSG | SPZ1 |
| 241 | NUCB2 | MMP13 |
| 242 | COX7C | WFDC11 |
| 243 | FNDC3B | SLCO1A2 |
| 244 | ATOX1 | ERICH3 |
| 245 | IFI30 | ATP6V0D2 |
| 246 | HNRNPK | SEL1L2 |
| 247 | SLC44A2 | SLC15A5 |
| 248 | THBS1 | OR2D3 |
| 249 | CD81 | XKR3 |
| 250 | ATP5ME | OR4K2 |
| 251 | PTTG1IP | ARL14 |
| 252 | TMEM258 | FMO1 |
| 253 | PDLIM1 | OR10H4 |
| 254 | YBX1 | TM4SF20 |
| 255 | TSKU | CR2 |
| 256 | PICALM | GYS2 |
| 257 | IL1B | SCG3 |
| 258 | KDELR1 | CPA2 |
| 259 | SH3GLB1 | LRRD1 |
| 260 | SPARCL1 | GABRA2 |
| 261 | EEF1G | TPD52L3 |
| 262 | PSMA7 | SMC1B |
| 263 | SERPINB2 | HAO1 |
| 264 | PKM | OR2A25 |
| 265 | CCL2 | DYNAP |
| 266 | IVNS1ABP | GRIA2 |
| 267 | ARL6IP1 | CFTR |
| 268 | ITGA5 | NHLH2 |
| 269 | CHCHD2 | OR8H3 |
| 270 | CHP1 | OR2T6 |
| 271 | TPP1 | OR6C6 |
| 272 | CD46 | ACOD1 |
| 273 | RPL36AL | OR5B3 |
| 274 | CSF3R | S100A7L2 |
| 275 | ARHGDIB | IL21 |
| 276 | NOTUM | MUC13 |
| 277 | S100A4 | MTTP |
| 278 | ERRFI1 | PTPRZ1 |
| 279 | RAB1B | OR6M1 |
| 280 | MYADM | DEPDC4 |
| 281 | SEC31A | PCDH15 |
| 282 | MXD1 | OR10A7 |
| 283 | EZR | SLC25A31 |
| 284 | ACTR2 | GADL1 |
| 285 | CXCL1 | RFX6 |
| 286 | FKBP1A | AK7 |
| 287 | SNRPD2 | GABRG1 |
| 288 | EFEMP1 | AFM |
| 289 | CD9 | ANO3 |
| 290 | ATP5PO | UGT2B4 |
| 291 | DAB2 | OR5D14 |
| 292 | TREM1 | OR4C3 |
| 293 | DDX5 | BAAT |
| 294 | ENG | TAT |
| 295 | S100A9 | TDRD1 |
| 296 | CTSD | PXT1 |
| 297 | ACTN1 | CDH9 |
| 298 | PMP22 | SPAM1 |
| 299 | RAB8B | RAD21L1 |
| 300 | FBN1 | RXRG |
| 301 | PAFAH1B2 | EPHA6 |
| 302 | ARRDC3 | OR1C1 |
| 303 | HTRA1 | SYNPR |
| 304 | CAPNS1 | C5orf47 |
| 305 | CNBP | C6orf58 |
| 306 | IGFBP5 | CFHR3 |
| 307 | PERP | GLIPR1L2 |
| 308 | PTMA | SPINT4 |
| 309 | ATP6V1E1 | SPACA1 |
| 310 | ACTG2 | FNDC7 |
| 311 | AHNAK | TUBAL3 |
| 312 | LCP2 | UGT3A1 |
| 313 | RBM39 | GPRC6A |
| 314 | RGS2 | CYP7A1 |
| 315 | SIGLEC6 | CFHR4 |
| 316 | IFI27 | GDF3 |
| 317 | RND3 | GSDMC |
| 318 | PI3 | FYB2 |
| 319 | FLT1 | CNTN3 |
| 320 | EGR1 | C6orf118 |
| 321 | HIF1A | TECTB |
| 322 | ERO1A | DCDC1 |
| 323 | ARL6IP5 | TFAP2D |
| 324 | B4GALT1 | TAAR6 |
| 325 | TM9SF2 | SULT1C4 |
| 326 | ERGIC3 | PLPPR5 |
| 327 | S100A6 | OR13G1 |
| 328 | EIF2S2 | LRP1B |
| 329 | C6orf62 | UTS2B |
| 330 | FNDC3A | PCDHB15 |
| 331 | FBLN1 | CUZD1 |
| 332 | CAST | PKD2L2 |
| 333 | MAT2A | XIRP2 |
| 334 | LGALS3 | POU1F1 |
| 335 | RPS5 | SLC9C2 |
| 336 | PNRC1 | EBLN1 |
| 337 | MTPN | DNAH14 |
| 338 | PCBP2 | SERPINI2 |
| 339 | TNFRSF1A | NPY4R |
| 340 | SF3B6 | TDRD5 |
| 341 | C1QB | OR4C15 |
| 342 | SSR1 | PRSS58 |
| 343 | C1QC | CYP24A1 |
| 344 | SRGN | GPR19 |
| 345 | SHISA5 | SLC9B1 |
| 346 | HIST1H1E | LRRC4C |
| 347 | TUBB4B | TACR3 |
| 348 | NFE2L2 | UGT2A1 |
| 349 | PROK1 | OR5H2 |
| 350 | FERMT2 | SLC25A21 |
| 351 | VEGFA | OR8I2 |
| 352 | AQP9 | RNASE12 |
| 353 | GNB1 | SLC26A3 |
| 354 | EIF4EBP2 | RAD9B |
| 355 | FXYD5 | CATSPERB |
| 356 | MAPK1IP1L | KRTAP19-7 |
| 357 | GNAI2 | TMEM225 |
| 358 | DPYSL2 | SLC17A1 |
| 359 | TRAM2 | MOGAT1 |
| 360 | CTNNB1 | ADAM18 |
| 361 | MAP1LC3B2 | AMBN |
| 362 | EPYC | DUSP19 |
| 363 | ATP2B4 | FSIP2 |
| 364 | F2R | FABP1 |
| 365 | CNN2 | EFCAB5 |
| 366 | ANP32B | CCDC173 |
| 367 | IL1RL1 | OR52J3 |
| 368 | LRPAP1 | MYO1H |
| 369 | TOMM7 | OR51A7 |
| 370 | ATP5MD | APOBEC4 |
| 371 | TPM2 | HTN3 |
| 372 | AOC1 | ENPP5 |
| 373 | PGRMC2 | EPHA7 |
| 374 | CALU | OR4X2 |
| 375 | JPT1 | NDST3 |
| 376 | PIGT | SULT2A1 |
| 377 | TRMT112 | ELAVL2 |
| 378 | RCN1 | TPH2 |
| 379 | IFNGR2 | SPP2 |
| 380 | ALOX5AP | SAMD12 |
| 381 | RHOBTB3 | C9orf135 |
| 382 | REEP5 | ASB15 |
| 383 | ITGB8 | C3orf30 |
| 384 | GATA3 | OR10A5 |
| 385 | ATP6V1B2 | TAAR8 |
| 386 | UGDH | ANKRD62 |
| 387 | VAMP3 | OR10J5 |
| 388 | ASAH1 | HAPLN1 |
| 389 | MDH2 | AKR1D1 |
| 390 | SEC61B | ADH6 |
| 391 | PLOD2 | OR2AP1 |
| 392 | MORF4L1 | OR5AS1 |
| 393 | ATP13A3 | HEPACAM2 |
| 394 | YWHAB | BOLL |
| 395 | EIF3K | SPIC |
| 396 | KIF5B | TYRP1 |
| 397 | STAT3 | C12orf50 |
| 398 | ANTXR1 | AFP |
| 399 | TNFAIP3 | TEX35 |
| 400 | AGFG1 | TMEM174 |
| 401 | TAGLN2 | TEX36 |
| 402 | TRAPPC1 | SNTN |
| 403 | ADAMTS1 | TAS2R16 |
| 404 | EFHD1 | NR2E1 |
| 405 | RAB5A | IFNB1 |
| 406 | CORO1C | C9orf152 |
| 407 | HILPDA | OR6C76 |
| 408 | SERPINA1 | TAS2R7 |
| 409 | CLTC | CLDN24 |
| 410 | EIF3D | DCSTAMP |
| 411 | UQCR11 | SUN3 |
| 412 | PPP1CB | MYOC |
| 413 | ARPC3 | POM121L2 |
| 414 | MBNL2 | SAMD13 |
| 415 | CYSTM1 | LIPC |
| 416 | ATP1A1 | PON1 |
| 417 | GDI2 | KRTAP3-3 |
| 418 | POMP | AMTN |
| 419 | HIST1H2AC | CNTN1 |
| 420 | CAPN2 | OR5T2 |
| 421 | PHLDB2 | NETO1 |
| 422 | TFPI | OLFM3 |
| 423 | ATP5F1C | NEUROD6 |
| 424 | SLC38A1 | IFNA2 |
| 425 | RBX1 | BMP10 |
| 426 | GCM1 | ADH7 |
| 427 | CD53 | PRR23C |
| 428 | UBQLN1 | BPIFC |
| 429 | TAX1BP1 | GPR85 |
| 430 | CHMP2A | PCDH9 |
| 431 | C3AR1 | STK32A |
| 432 | ETS2 | OR5AC2 |
| 433 | MYOF | WDR72 |
| 434 | BZW2 | ANGPTL5 |
| 435 | SNX10 | INSL5 |
| 436 | PCBP1 | OR4C16 |
| 437 | ITGA6 | OR1J1 |
| 438 | AFF1 | SEMG2 |
| 439 | BTG2 | AKR1C4 |
| 440 | CSNK1A1 | SLC5A7 |
| 441 | PSMD8 | SYT14 |
| 442 | RAP1B | CNPY1 |
| 443 | TUBB2A | SLITRK3 |
| 444 | TGFBI | SNTG1 |
| 445 | SEC63 | GC |
| 446 | RPS7 | GLYATL1 |
| 447 | JAK1 | CADPS |
| 448 | LITAF | OR4D2 |
| 449 | CAPZB | DGKB |
| 450 | EIF3H | IL4 |
| 451 | CALD1 | CLCA4 |
| 452 | DSP | SLC17A6 |
| 453 | HMGN1 | GABRB1 |
| 454 | LSM14A | DSG4 |
| 455 | EMP3 | FOXP2 |
| 456 | HNRNPA3 | MYH4 |
| 457 | CAV1 | RBM44 |
| 458 | SDC4 | OR56A5 |
| 459 | ADAMTS5 | TMPRSS11F |
| 460 | PLPP3 | CLEC2A |
| 461 | EIF4B | YIPF7 |
| 462 | C15orf48 | PRAC1 |
| 463 | HMOX1 | IQUB |
| 464 | NDUFB4 | EXD1 |
| 465 | CRISPLD2 | DAO |
| 466 | CPXM1 | SAMD7 |
| 467 | ERBIN | SPDYA |
| 468 | TCP1 | SPOCK3 |
| 469 | TRAM1 | LYZL2 |
| 470 | SSB | RERGL |
| 471 | FLNB | POLQ |
| 472 | STAT1 | DNAH3 |
| 473 | HK2 | KLHL32 |
| 474 | LMAN1 | FREM3 |
| 475 | ALDOA | PRSS55 |
| 476 | LASP1 | FUT9 |
| 477 | GLIPR1 | HHLA2 |
| 478 | RYBP | REC114 |
| 479 | IFNGR1 | TMC2 |
| 480 | GBP3 | OR4C11 |
| 481 | PARVA | KRTAP13-3 |
| 482 | AFF4 | MCMDC2 |
| 483 | BZW1 | KCNH5 |
| 484 | CHMP5 | OR10A6 |
| 485 | CDA | ART3 |
| 486 | INHBA | OR13C3 |
| 487 | ELL2 | TAAR2 |
| 488 | NRIP1 | HIST1H2BA |
| 489 | YAP1 | GKN2 |
| 490 | COL6A3 | ADAM30 |
| 491 | AP2M1 | CPLX4 |
| 492 | SDCBP | PLCZ1 |
| 493 | ELF1 | GRM7 |
| 494 | DNAJC3 | GABRG3 |
| 495 | HSPD1 | GTSF1L |
| 496 | VCAN | IL20 |
| 497 | PROK2 | HOXC5 |
| 498 | ESYT2 | GLRA3 |
| 499 | LTBP1 | RFX8 |
| 500 | ADAM10 | RGS21 |

Top500 and bottom500 genes are based on the RNA-Seq data of the placental tissues from the preterm pregnancies in GSE73685.
