# Supplementary material for: Genome-wide nucleosome footprints of plasma cfDNA predict preterm birth: A case-control study
Source: PLoS Med. 2025 Apr 15;22(4):e1004571. doi: 10.1371/journal.pmed.1004571 (PMC11999135; doi:10.1371/journal.pmed.1004571)
Supplement: S2 Table — (DOCX) [file pmed.1004571.s009.docx]

**S2 Table. Placenta- and whole blood-enriched genes**

| **Placenta-enriched Genes** | **Blood-enriched Genes** |
| --- | --- |
| CSH1 | ABHD5 |
| CSH2 | ABTB1 |
| TFPI2 | ACSL1 |
| KISS1 | ADAM8 |
| PSG3 | AIF1 |
| PAPPA | AMICA1 |
| PSG4 | AOAH |
| CYP19A1 | AP1S2 |
| ADAM12 | APOBEC3A |
| PSG2 | APOBR |
| PSG1 | AQP9 |
| PSG9 | ARAP1 |
| HSD3B1 | ARHGAP25 |
| EBI3 | ARHGAP26 |
| PAPPA2 | ARHGAP30 |
| PSG11 | ARRB2 |
| ALPP | BACH1 |
| PSG5 | BAZ1A |
| CSHL1 | BCL2A1 |
| GH2 | BID |
| PSG6 | BIN2 |
| CRH | C10orf54 |
| PSG7 | C15orf39 |
| CAPN6 | C1orf162 |
| HBG2 | C1orf38 |
| MMP11 | C21orf7 |
| PSG8 | C5AR1 |
| PLAC1 | CASP1 |
| ERVW-1 | CASP4 |
| XAGE2 | CASP8 |
| SIGLEC6 | CCL5 |
| LGALS14 | CCR1 |
| HSD17B1 | CCR2 |
| XAGE3 | CD300A |
| PAGE4 | CD302 |
| GCM1 | CD58 |
| LGALS13 | CD97 |
| CGB5 | CDC42SE1 |
| CGB8 | CFP |
| VGLL1 | CHST15 |
| ISM2 | CKLF |
| INSL4 | CLEC2B |
| CGB3 | CLEC4A |
| TRIM40 | CLEC7A |
| LIN28B | CORO1A |
| ZNF593 | CRLF3 |
| ERVFRD-1 | CSF2RB |
| HTRA4 | CSF3R |
| SLC6A2 | CTBS |
| ERVV-2 | CTSS |
| ERVV-1 | CTSW |
| MAGEA10 | CXCR1 |
| MAGEA8 | CXCR2 |
| LGALS16 | CYB5R4 |
| H2BFS | CYBB |
| ANKRD33 | CYTH4 |
| NPFFR2 | CYTIP |
| GNGT1 | DGAT2 |
| ZBTB9 | DPEP2 |
| BLID | EHBP1L1 |
| ALPPL2 | EMR2 |
| DSCR4 | EMR3 |
| AMIGO3 | EVI2B |
| RNASE12 | FAM129A |
| GPR21 | FAM63A |
| OR9A2 | FAM65B |
| MTNR1B | FAS |
| TAS2R41 | FCER1G |
| TXNRD3NB | FCGR2A |
| OR52E6 | FCGR3B |
| OR52E8 | FCN1 |
| / | FERMT3 |
| / | FFAR2 |
| / | FGD3 |
| / | FGL2 |
| / | FGR |
| / | FLI1 |
| / | FMNL1 |
| / | FOLR3 |
| / | FPR1 |
| / | FPR2 |
| / | FRAT1 |
| / | FRAT2 |
| / | FYB |
| / | GBP2 |
| / | GBP5 |
| / | GCA |
| / | GIMAP4 |
| / | GMFG |
| / | GMIP |
| / | GNLY |
| / | GPSM3 |
| / | GZMA |
| / | GZMB |
| / | GZMH |
| / | HAUS4 |
| / | HCAR3 |
| / | HCK |
| / | HCLS1 |
| / | HCP5 |
| / | HIST1H3H |
| / | HSPA6 |
| / | ICAM3 |
| / | IFIT2 |
| / | IGSF6 |
| / | IL10RA |
| / | IL18RAP |
| / | IL6R |
| / | IRF1 |
| / | IRF2 |
| / | ITGA2B |
| / | ITGB2 |
| / | KCNJ2 |
| / | KLRB1 |
| / | LAPTM5 |
| / | LAT2 |
| / | LCP1 |
| / | LCP2 |
| / | LILRA1 |
| / | LILRA2 |
| / | LILRA3 |
| / | LILRA5 |
| / | LILRA6 |
| / | LILRB2 |
| / | LIMD2 |
| / | LRRFIP1 |
| / | LSP1 |
| / | LST1 |
| / | LY96 |
| / | LYN |
| / | LYST |
| / | MAPK14 |
| / | MEGF9 |
| / | MLH3 |
| / | MNDA |
| / | MS4A6A |
| / | MSRB1 |
| / | MX2 |
| / | MYD88 |
| / | MYO1F |
| / | N4BP1 |
| / | NADK |
| / | NAGA |
| / | NAMPT |
| / | NCF2 |
| / | NCF4 |
| / | NFE2 |
| / | NKG7 |
| / | NMI |
| / | NOD2 |
| / | NPL |
| / | NRBF2 |
| / | NUMB |
| / | OSBPL11 |
| / | OSTF1 |
| / | P2RY13 |
| / | PAK2 |
| / | PF4 |
| / | PF4V1 |
| / | PIK3CD |
| / | PILRA |
| / | PLBD1 |
| / | PLEK |
| / | PLXNC1 |
| / | PPBP |
| / | PRKCD |
| / | PROK2 |
| / | PSMB9 |
| / | PSTPIP1 |
| / | PTEN |
| / | PTPN6 |
| / | PTPRC |
| / | PTPRE |
| / | PXN |
| / | PYGL |
| / | R3HDM4 |
| / | RARA |
| / | RASSF5 |
| / | RCBTB2 |
| / | RCSD1 |
| / | RGS19 |
| / | RGS2 |
| / | RNASE6 |
| / | S100A4 |
| / | S1PR4 |
| / | SEC14L1 |
| / | SECTM1 |
| / | SELL |
| / | SELPLG |
| / | SKAP2 |
| / | SMCHD1 |
| / | SP110 |
| / | SPI1 |
| / | STK10 |
| / | STK38 |
| / | STX3 |
| / | STXBP2 |
| / | TAGAP |
| / | TLE3 |
| / | TLR1 |
| / | TLR2 |
| / | TLR8 |
| / | TMEM140 |
| / | TMEM71 |
| / | TNFAIP2 |
| / | TNFRSF10C |
| / | TNFRSF1B |
| / | TNFSF10 |
| / | TREM1 |
| / | TRIM21 |
| / | TYROBP |
| / | UBE2D1 |
| / | USP15 |
| / | VNN2 |
| / | WAS |
| / | XPO6 |
| / | ZCCHC6 |
| / | ZDHHC18 |
| / | ZNF267 |
| / | ZNF467 |

Placenta- and blood-enriched genes were obtained from the study of Gong *et al*, 2021, *Nature Communications* and PaGenBase.
