# Supplementary material for: Genome-wide nucleosome footprints of plasma cfDNA predict preterm birth: A case-control study
Source: PLoS Med. 2025 Apr 15;22(4):e1004571. doi: 10.1371/journal.pmed.1004571 (PMC11999135; doi:10.1371/journal.pmed.1004571)
Supplement: S4 Table — (DOCX) [file pmed.1004571.s011.docx]

**S4 Table. Clinical characteristics of pregnancies in discovery cohort**

| Characteristics | Preterm (n=20) | Full-term (n=20) | *P-*value |
| --- | --- | --- | --- |
| Gestational age at sampling (weeks) | 15.7+4.3 | 15.3+3.0 | 0.386 |
| Maternal age (years) | 30.6+4.9 | 30.1+2.6 | 0.946 |
| BMI (kg/m^2^) | 21.0+2.5 | 20.9+3.6 | 0.626 |

Data are mean ± standard deviation. Age = maternal age. BMI = pre-pregnancy body mass index. Two-sided Wilcoxon rank-sum test was used for the comparison of continuous variables (n=40, 20 preterm and 20 full-term pregnancies).
