# Supplementary material for: Genome-wide nucleosome footprints of plasma cfDNA predict preterm birth: A case-control study
Source: PLoS Med. 2025 Apr 15;22(4):e1004571. doi: 10.1371/journal.pmed.1004571 (PMC11999135; doi:10.1371/journal.pmed.1004571)
Supplement: S5 Table — (DOCX) [file pmed.1004571.s012.docx]

**S5 Table. Genes with differential read coverages at the pTSS**

| RefSeq | GeneSymbol | MeanPreterm | MeanFullterm | FoldChange | FDR | Rank | Retain |
| --- | --- | --- | --- | --- | --- | --- | --- |
| NM_001318170 | MPP7 | 12.27 | 24.89 | 0.49 | 0.0427 | 111 | 1 |
| NM_003174 | SVIL | 12.14 | 30.03 | 0.40 | 0.0223 | 77 | 1 |
| NM_001323600 | SVIL | 11.56 | 28.80 | 0.40 | 0.0212 | 78 | 0 |
| NM_001143769 | ZNF438 | 20.51 | 41.49 | 0.49 | 0.0203 | 195 | 1 |
| NM_025209 | EPC1 | 18.02 | 6.97 | 2.58 | 0.0481 | 192 | 1 |
| NM_001098206 | HNRNPF | 10.61 | 22.87 | 0.46 | 0.0203 | 254 | 1 |
| NM_001242413 | PRKCQ | 34.59 | 14.33 | 2.41 | 0.0191 | 101 | 1 |
| NM_001323265 | PRKCQ | 32.09 | 12.75 | 2.52 | 0.0201 | 157 | 0 |
| NM_017516 | RAB39A | 24.98 | 12.39 | 2.02 | 0.0332 | 172 | 1 |
| NM_001271983 | C11orf44 | 19.65 | 39.59 | 0.50 | 0.0171 | 156 | 1 |
| NM_003594 | TTF2 | 9.64 | 23.40 | 0.41 | 0.0163 | 203 | 1 |
| NM_153347 | TMEM86A | 28.68 | 13.89 | 2.06 | 0.0285 | 188 | 1 |
| NM_001142315 | LMO2 | 25.53 | 12.69 | 2.01 | 0.0479 | 215 | 1 |
| NM_005574 | LMO2 | 15.99 | 32.23 | 0.50 | 0.0246 | 131 | 1 |
| NM_001270550 | MDK | 33.41 | 15.70 | 2.13 | 0.0244 | 135 | 0 |
| NM_001012334 | MDK | 31.10 | 14.80 | 2.10 | 0.0218 | 133 | 1 |
| NM_001258289 | SELENBP1 | 35.77 | 17.33 | 2.06 | 0.0365 | 48 | 1 |
| NM_020770 | CGN | 32.31 | 15.99 | 2.02 | 0.0167 | 224 | 1 |
| NM_001010979 | C1orf189 | 20.40 | 42.91 | 0.48 | 0.0163 | 66 | 1 |
| NM_006912 | RIT1 | 29.67 | 13.43 | 2.21 | 0.0203 | 223 | 1 |
| NM_152716 | PATL1 | 10.39 | 21.88 | 0.47 | 0.0454 | 26 | 1 |
| NM_207340 | ZDHHC24 | 11.41 | 25.04 | 0.46 | 0.0440 | 57 | 0 |
| NM_001258371 | ACTN3 | 13.13 | 27.98 | 0.47 | 0.0227 | 55 | 1 |
| NM_001104 | ACTN3 | 12.72 | 31.51 | 0.40 | 0.0163 | 112 | 0 |
| NM_001256405 | KDM2A | 30.66 | 12.91 | 2.37 | 0.0187 | 88 | 1 |
| NM_005608 | PTPRCAP | 29.02 | 11.50 | 2.52 | 0.0200 | 14 | 1 |
| NM_212469 | CHKA | 19.44 | 6.22 | 3.13 | 0.0313 | 110 | 1 |
| NM_014824 | FCHSD2 | 20.69 | 10.18 | 2.03 | 0.0312 | 98 | 1 |
| NM_138705 | CALML6 | 15.15 | 33.65 | 0.45 | 0.0163 | 252 | 1 |
| NM_001286094 | TMEM9B | 21.36 | 9.13 | 2.34 | 0.0363 | 106 | 1 |
| NM_005475 | SH2B3 | 22.10 | 9.43 | 2.34 | 0.0481 | 95 | 1 |
| NM_001030287 | ATF3 | 20.76 | 41.82 | 0.50 | 0.0201 | 125 | 1 |
| NM_003268 | TLR5 | 25.64 | 11.63 | 2.20 | 0.0203 | 189 | 1 |
| NM_005426 | TP53BP2 | 19.76 | 6.23 | 3.17 | 0.0163 | 225 | 1 |
| NM_033631 | LUZP1 | 31.51 | 15.23 | 2.07 | 0.0163 | 127 | 1 |
| NM_001098576 | TMBIM6 | 10.59 | 21.63 | 0.49 | 0.0256 | 218 | 1 |
| NM_001352322 | LARP4 | 32.75 | 12.94 | 2.53 | 0.0203 | 147 | 1 |
| NM_001352318 | LARP4 | 33.03 | 15.61 | 2.12 | 0.0384 | 162 | 0 |
| NM_182507 | KRT80 | 18.83 | 39.02 | 0.48 | 0.0201 | 261 | 1 |
| NM_134324 | TARBP2 | 5.54 | 14.17 | 0.39 | 0.0383 | 114 | 1 |
| NM_001002031 | ATP5MC2 | 22.33 | 10.49 | 2.13 | 0.0358 | 105 | 1 |
| NM_006897 | HOXC9 | 30.89 | 14.10 | 2.19 | 0.0365 | 103 | 1 |
| NM_001113201 | NACA | 20.71 | 8.66 | 2.39 | 0.0203 | 23 | 1 |
| NM_001320193 | NACA | 19.87 | 7.36 | 2.70 | 0.0163 | 269 | 0 |
| NM_138396 | MARCHF9 | 12.41 | 25.80 | 0.48 | 0.0201 | 100 | 1 |
| NM_001320410 | ATP23 | 9.05 | 18.95 | 0.48 | 0.0422 | 242 | 1 |
| NM_001135734 | ZNF384 | 8.67 | 17.51 | 0.50 | 0.0457 | 231 | 1 |
| NM_001297643 | C1RL | 10.08 | 28.83 | 0.35 | 0.0270 | 163 | 1 |
| NM_005248 | FGR | 36.22 | 17.18 | 2.11 | 0.0363 | 44 | 1 |
| NM_001166007 | EPB41 | 35.48 | 17.49 | 2.03 | 0.0457 | 32 | 1 |
| NM_003566 | EEA1 | 10.45 | 23.06 | 0.45 | 0.0167 | 150 | 1 |
| NM_007129 | ZIC2 | 37.06 | 14.61 | 2.54 | 0.0176 | 9 | 1 |
| NM_005537 | ING1 | 18.89 | 8.26 | 2.29 | 0.0227 | 183 | 0 |
| NM_001267728 | ING1 | 21.10 | 7.78 | 2.71 | 0.0182 | 113 | 1 |
| NM_015205 | ATP11A | 26.75 | 11.57 | 2.31 | 0.0334 | 140 | 1 |
| NM_001014283 | DCUN1D2 | 18.25 | 8.27 | 2.21 | 0.0339 | 10 | 0 |
| NM_001349742 | TMCO3 | 18.31 | 8.27 | 2.21 | 0.0355 | 8 | 1 |
| NM_018134 | IQCC | 11.42 | 28.19 | 0.41 | 0.0214 | 180 | 1 |
| NM_023009 | MARCKSL1 | 24.39 | 11.62 | 2.10 | 0.0292 | 276 | 1 |
| NM_012158 | FBXL3 | 17.18 | 37.23 | 0.46 | 0.0201 | 211 | 1 |
| NM_001040153 | SLAIN1 | 34.03 | 15.26 | 2.23 | 0.0423 | 21 | 1 |
| NM_001350765 | C1orf109 | 18.75 | 8.77 | 2.14 | 0.0478 | 40 | 0 |
| NM_001256875 | CDCA8 | 18.20 | 8.66 | 2.10 | 0.0493 | 39 | 1 |
| NM_001198983 | TEDC1 | 7.12 | 19.01 | 0.37 | 0.0167 | 73 | 1 |
| NM_001134877 | TEDC1 | 5.34 | 17.63 | 0.30 | 0.0167 | 161 | 0 |
| NM_001291281 | FOXO6 | 25.69 | 10.61 | 2.42 | 0.0440 | 121 | 1 |
| NM_033400 | ZFHX2 | 11.92 | 27.78 | 0.43 | 0.0163 | 151 | 1 |
| NM_014430 | CIDEB | 17.21 | 8.14 | 2.11 | 0.0440 | 191 | 0 |
| NM_001318807 | CIDEB | 17.21 | 8.14 | 2.11 | 0.0440 | 47 | 1 |
| NM_001198965 | NFATC4 | 27.58 | 12.67 | 2.18 | 0.0309 | 124 | 1 |
| NM_020529 | NFKBIA | 8.56 | 20.53 | 0.42 | 0.0244 | 58 | 1 |
| NM_020692 | GALNT16 | 27.22 | 12.29 | 2.21 | 0.0339 | 142 | 1 |
| NM_001080414 | CCDC88C | 7.03 | 26.55 | 0.26 | 0.0191 | 253 | 1 |
| NM_001278631 | GABRB3 | 13.92 | 34.21 | 0.41 | 0.0201 | 35 | 1 |
| NM_021912 | GABRB3 | 14.38 | 34.77 | 0.41 | 0.0214 | 36 | 0 |
| NM_017553 | INO80 | 10.14 | 22.87 | 0.44 | 0.0420 | 256 | 1 |
| NM_020759 | STARD9 | 8.68 | 19.71 | 0.44 | 0.0355 | 136 | 1 |
| NM_001316935 | HSPB11 | 19.23 | 7.46 | 2.58 | 0.0365 | 30 | 1 |
| NM_052940 | LRRC42 | 19.23 | 7.01 | 2.74 | 0.0358 | 31 | 0 |
| NM_001025249 | DUT | 7.44 | 20.78 | 0.36 | 0.0323 | 139 | 1 |
| NM_152450 | FAM81A | 13.13 | 29.39 | 0.45 | 0.0214 | 43 | 1 |
| NM_130434 | DPP8 | 20.38 | 8.90 | 2.29 | 0.0203 | 264 | 1 |
| NM_001324092 | GLCE | 22.38 | 11.01 | 2.03 | 0.0201 | 178 | 1 |
| NM_001286679 | LARP6 | 18.13 | 38.29 | 0.47 | 0.0228 | 65 | 1 |
| NM_001352019 | LMF1 | 31.83 | 13.66 | 2.33 | 0.0182 | 12 | 0 |
| NM_014587 | SOX8 | 35.54 | 16.00 | 2.22 | 0.0203 | 5 | 1 |
| NM_001256160 | ATF7IP2 | 27.72 | 11.40 | 2.43 | 0.0227 | 220 | 1 |
| NM_002738 | PRKCB | 10.44 | 21.12 | 0.49 | 0.0306 | 176 | 1 |
| NM_016333 | SRRM2 | 27.81 | 8.43 | 3.30 | 0.0176 | 235 | 1 |
| NM_001142448 | SPNS1 | 13.85 | 30.56 | 0.45 | 0.0256 | 144 | 1 |
| NM_001324494 | ZNF48 | 28.25 | 12.94 | 2.18 | 0.0306 | 34 | 1 |
| NM_024671 | ZNF768 | 9.09 | 22.46 | 0.40 | 0.0176 | 61 | 1 |
| NM_001142544 | CTF1 | 9.65 | 24.73 | 0.39 | 0.0203 | 81 | 1 |
| NM_001136505 | TERB1 | 15.69 | 31.67 | 0.50 | 0.0201 | 277 | 1 |
| NM_016062 | FAM96B | 13.89 | 28.01 | 0.50 | 0.0479 | 274 | 0 |
| NM_003869 | CES2 | 13.89 | 28.01 | 0.50 | 0.0479 | 229 | 1 |
| NM_001040715 | KIAA0895L | 10.56 | 23.88 | 0.44 | 0.0334 | 174 | 1 |
| NM_001352278 | RHOT2 | 19.67 | 9.42 | 2.09 | 0.0283 | 134 | 1 |
| NM_022041 | GAN | 18.21 | 37.06 | 0.49 | 0.0256 | 166 | 1 |
| NM_001171815 | RNF166 | 37.20 | 16.51 | 2.25 | 0.0191 | 16 | 1 |
| NM_178841 | RNF166 | 36.73 | 17.02 | 2.16 | 0.0203 | 238 | 0 |
| NM_001318507 | CTU2 | 35.12 | 17.02 | 2.06 | 0.0244 | 29 | 0 |
| NM_153321 | PMP22 | 15.79 | 32.48 | 0.49 | 0.0256 | 137 | 1 |
| NM_020889 | PHF12 | 11.19 | 4.48 | 2.50 | 0.0397 | 71 | 1 |
| NM_033419 | PGAP3 | 11.38 | 25.19 | 0.45 | 0.0431 | 267 | 0 |
| NM_001289936 | ERBB2 | 11.38 | 25.19 | 0.45 | 0.0431 | 160 | 1 |
| NM_002809 | PSMD3 | 10.88 | 23.32 | 0.47 | 0.0281 | 249 | 1 |
| NM_001190919 | THRA | 25.86 | 12.45 | 2.08 | 0.0270 | 193 | 0 |
| NM_003250 | THRA | 23.23 | 8.19 | 2.84 | 0.0331 | 83 | 1 |
| NM_017643 | MBTD1 | 6.29 | 15.40 | 0.41 | 0.0479 | 266 | 1 |
| NM_001193611 | TUBD1 | 8.69 | 25.71 | 0.34 | 0.0171 | 67 | 0 |
| NM_001272042 | RPS6KB1 | 7.97 | 24.00 | 0.33 | 0.0171 | 54 | 1 |
| NM_001330431 | MAP3K3 | 23.21 | 10.80 | 2.15 | 0.0356 | 4 | 0 |
| NM_203351 | MAP3K3 | 22.15 | 10.80 | 2.05 | 0.0470 | 3 | 1 |
| NM_000891 | KCNJ2 | 23.29 | 8.27 | 2.81 | 0.0270 | 52 | 1 |
| NM_201566 | SLC16A13 | 22.68 | 8.02 | 2.83 | 0.0163 | 49 | 1 |
| NM_024297 | PHF23 | 17.23 | 5.63 | 3.06 | 0.0218 | 63 | 1 |
| NM_182565 | UBALD2 | 19.53 | 8.75 | 2.23 | 0.0256 | 24 | 1 |
| NM_001349618 | CAMTA1 | 9.03 | 21.08 | 0.43 | 0.0254 | 202 | 0 |
| NM_001349626 | CAMTA1 | 9.03 | 21.05 | 0.43 | 0.0256 | 201 | 0 |
| NM_001349615 | CAMTA1 | 9.03 | 21.05 | 0.43 | 0.0256 | 116 | 0 |
| NM_001349619 | CAMTA1 | 9.03 | 21.05 | 0.43 | 0.0256 | 109 | 0 |
| NM_001349616 | CAMTA1 | 9.50 | 20.51 | 0.46 | 0.0365 | 108 | 1 |
| NM_001349622 | CAMTA1 | 9.50 | 20.51 | 0.46 | 0.0365 | 214 | 0 |
| NM_019020 | TBC1D16 | 29.88 | 12.98 | 2.30 | 0.0222 | 232 | 1 |
| NM_004920 | AATK | 33.86 | 15.96 | 2.12 | 0.0214 | 200 | 1 |
| NM_001277333 | ANKRD62 | 10.63 | 21.63 | 0.49 | 0.0481 | 260 | 1 |
| NM_018030 | OSBPL1A | 15.05 | 31.14 | 0.48 | 0.0163 | 74 | 1 |
| NM_001258222 | KCTD1 | 13.77 | 27.93 | 0.49 | 0.0406 | 41 | 1 |
| NM_001168335 | ME2 | 12.70 | 28.14 | 0.45 | 0.0358 | 115 | 1 |
| NM_001243702 | ZBTB14 | 6.26 | 14.11 | 0.44 | 0.0334 | 272 | 1 |
| NM_001146345 | PQLC1 | 24.09 | 11.87 | 2.03 | 0.0481 | 27 | 1 |
| NM_001300815 | CIRBP | 7.02 | 20.41 | 0.34 | 0.0457 | 104 | 0 |
| NM_001280 | CIRBP | 7.02 | 20.41 | 0.34 | 0.0457 | 87 | 1 |
| NM_004461 | FARSA | 8.84 | 19.83 | 0.45 | 0.0193 | 219 | 1 |
| NM_001271611 | STX10 | 18.58 | 8.41 | 2.21 | 0.0212 | 85 | 0 |
| NM_004907 | IER2 | 18.64 | 8.41 | 2.22 | 0.0205 | 60 | 1 |
| NM_001128855 | GTPBP3 | 10.58 | 22.86 | 0.46 | 0.0336 | 56 | 1 |
| NM_004386 | NCAN | 14.50 | 29.78 | 0.49 | 0.0256 | 187 | 1 |
| NM_001278575 | THEG5 | 17.91 | 41.22 | 0.43 | 0.0163 | 227 | 1 |
| NM_000969 | RPL5 | 29.40 | 14.49 | 2.03 | 0.0214 | 155 | 1 |
| NM_003419 | ZNF345 | 24.19 | 11.85 | 2.04 | 0.0201 | 210 | 0 |
| NM_001242800 | ZNF790 | 23.34 | 10.94 | 2.13 | 0.0204 | 258 | 0 |
| NM_001242474 | ZNF345 | 22.88 | 10.94 | 2.09 | 0.0214 | 175 | 1 |
| NM_182704 | SELENOV | 13.64 | 30.43 | 0.45 | 0.0171 | 84 | 1 |
| NM_001330519 | ZNF574 | 34.50 | 15.77 | 2.19 | 0.0163 | 182 | 1 |
| NM_001080400 | PLIN4 | 40.20 | 16.51 | 2.44 | 0.0171 | 217 | 1 |
| NM_202001 | ERCC1 | 26.64 | 11.83 | 2.25 | 0.0214 | 205 | 1 |
| NM_152362 | TNFAIP8L1 | 11.46 | 23.25 | 0.49 | 0.0481 | 53 | 1 |
| NM_001328666 | ARHGAP29 | 31.92 | 13.62 | 2.34 | 0.0171 | 270 | 1 |
| NM_145056 | DACT3 | 23.23 | 10.59 | 2.19 | 0.0468 | 245 | 1 |
| NM_178511 | INAFM1 | 18.12 | 6.77 | 2.68 | 0.0443 | 196 | 1 |
| NM_001277075 | ZNF541 | 25.78 | 12.05 | 2.14 | 0.0415 | 199 | 1 |
| NM_020719 | PRR12 | 26.68 | 9.46 | 2.82 | 0.0223 | 263 | 1 |
| NM_006270 | RRAS | 16.92 | 8.20 | 2.06 | 0.0479 | 82 | 1 |
| NM_001316994 | SPACA6 | 21.94 | 10.33 | 2.12 | 0.0241 | 167 | 1 |
| NM_032701 | KMT5C | 14.10 | 5.64 | 2.50 | 0.0479 | 207 | 1 |
| NM_001195259 | TGFBR3L | 12.37 | 24.90 | 0.50 | 0.0470 | 19 | 1 |
| NM_022482 | GZF1 | 24.94 | 8.76 | 2.85 | 0.0200 | 99 | 1 |
| NM_001008409 | TTLL9 | 18.63 | 38.09 | 0.49 | 0.0163 | 28 | 1 |
| NM_003098 | SNTA1 | 19.53 | 9.46 | 2.06 | 0.0256 | 259 | 1 |
| NM_006690 | MMP24 | 11.36 | 26.19 | 0.43 | 0.0427 | 149 | 1 |
| NM_021248 | CDH22 | 32.05 | 15.36 | 2.09 | 0.0363 | 129 | 1 |
| NM_005985 | SNAI1 | 10.97 | 24.50 | 0.45 | 0.0244 | 145 | 1 |
| NM_080821 | FAM210B | 11.52 | 26.55 | 0.43 | 0.0191 | 68 | 1 |
| NM_080622 | ABHD16B | 12.74 | 28.43 | 0.45 | 0.0203 | 244 | 1 |
| NM_001172646 | PLCB4 | 14.23 | 32.19 | 0.44 | 0.0201 | 222 | 1 |
| NM_007038 | ADAMTS5 | 13.63 | 29.70 | 0.46 | 0.0365 | 117 | 1 |
| NM_001350062 | TANC1 | 13.15 | 27.07 | 0.49 | 0.0476 | 90 | 1 |
| NM_001313904 | NFE2L2 | 27.93 | 12.05 | 2.32 | 0.0363 | 194 | 1 |
| NM_006164 | NFE2L2 | 32.01 | 14.86 | 2.15 | 0.0202 | 241 | 0 |
| NM_001042519 | C2orf88 | 37.74 | 16.42 | 2.30 | 0.0163 | 93 | 1 |
| NM_032321 | C2orf88 | 35.07 | 16.54 | 2.12 | 0.0163 | 94 | 0 |
| NM_001321691 | BZW1 | 12.13 | 24.40 | 0.50 | 0.0315 | 165 | 0 |
| NM_001207069 | BZW1 | 10.86 | 28.15 | 0.39 | 0.0163 | 7 | 0 |
| NM_001207068 | BZW1 | 10.36 | 27.62 | 0.38 | 0.0163 | 6 | 1 |
| NM_130906 | PPIL3 | 25.35 | 11.26 | 2.25 | 0.0163 | 185 | 0 |
| NM_001142355 | NIF3L1 | 24.90 | 10.80 | 2.31 | 0.0163 | 11 | 1 |
| NM_021824 | NIF3L1 | 26.13 | 10.67 | 2.45 | 0.0163 | 45 | 0 |
| NM_021141 | XRCC5 | 23.42 | 9.72 | 2.41 | 0.0202 | 153 | 1 |
| NM_001206878 | CTDSP1 | 23.45 | 11.69 | 2.01 | 0.0256 | 247 | 1 |
| NM_030573 | THAP7 | 16.88 | 6.99 | 2.42 | 0.0494 | 221 | 1 |
| NM_198440 | DERL3 | 15.08 | 6.27 | 2.41 | 0.0423 | 204 | 1 |
| NM_020437 | ASPHD2 | 38.11 | 14.30 | 2.66 | 0.0208 | 42 | 1 |
| NM_014306 | RTCB | 13.00 | 28.45 | 0.46 | 0.0228 | 50 | 1 |
| NM_206895 | SNORC | 15.13 | 35.07 | 0.43 | 0.0363 | 118 | 1 |
| NM_145640 | APOL3 | 17.81 | 37.85 | 0.47 | 0.0163 | 209 | 1 |
| NM_001349315 | SEPTIN2 | 7.41 | 17.55 | 0.42 | 0.0428 | 268 | 0 |
| NM_005336 | HDLBP | 4.13 | 19.56 | 0.21 | 0.0163 | 159 | 0 |
| NM_001282972 | SEPTIN2 | 4.13 | 19.00 | 0.22 | 0.0163 | 20 | 1 |
| NM_001349290 | SEPTIN2 | 7.51 | 21.39 | 0.35 | 0.0239 | 181 | 0 |
| NM_001008779 | SPDYA | 46.68 | 22.68 | 2.06 | 0.0129 | 228 | 1 |
| NM_000104 | CYP1B1 | 12.70 | 27.42 | 0.46 | 0.0256 | 92 | 1 |
| NM_138370 | PKDCC | 11.20 | 24.50 | 0.46 | 0.0398 | 248 | 1 |
| NM_001288953 | TTC7A | 20.63 | 8.97 | 2.30 | 0.0457 | 62 | 1 |
| NM_001288951 | TTC7A | 21.06 | 7.66 | 2.75 | 0.0220 | 243 | 1 |
| NM_004161 | RAB1A | 8.57 | 20.48 | 0.42 | 0.0214 | 76 | 1 |
| NM_178439 | GMCL1 | 33.88 | 15.01 | 2.26 | 0.0208 | 72 | 1 |
| NM_020459 | PAIP2B | 12.44 | 26.58 | 0.47 | 0.0203 | 123 | 1 |
| NM_032319 | PRADC1 | 18.44 | 7.71 | 2.39 | 0.0244 | 237 | 1 |
| NM_001009899 | USF3 | 21.47 | 9.02 | 2.38 | 0.0479 | 119 | 1 |
| NM_002213 | ITGB5 | 7.63 | 21.46 | 0.36 | 0.0495 | 33 | 1 |
| NM_001354690 | RAF1 | 13.91 | 27.88 | 0.50 | 0.0479 | 234 | 1 |
| NM_005862 | STAG1 | 18.02 | 5.17 | 3.48 | 0.0363 | 46 | 1 |
| NM_018155 | SLC25A36 | 12.64 | 25.84 | 0.49 | 0.0445 | 89 | 1 |
| NM_001349279 | ANKRD28 | 16.99 | 7.23 | 2.35 | 0.0339 | 170 | 0 |
| NM_001308036 | NCBP2 | 26.81 | 7.77 | 3.45 | 0.0129 | 13 | 1 |
| NM_007362 | NCBP2 | 25.23 | 8.45 | 2.99 | 0.0163 | 186 | 0 |
| NM_001355243 | NCBP2-AS2 | 25.74 | 8.95 | 2.88 | 0.0167 | 120 | 1 |
| NM_002673 | PLXNB1 | 30.16 | 14.06 | 2.15 | 0.0339 | 59 | 1 |
| NM_001200029 | HYAL3 | 25.19 | 10.45 | 2.41 | 0.0200 | 251 | 0 |
| NM_001200018 | NAT6 | 24.61 | 10.45 | 2.35 | 0.0201 | 250 | 1 |
| NM_018398 | CACNA2D3 | 34.40 | 11.44 | 3.01 | 0.0283 | 128 | 1 |
| NM_003392 | WNT5A | 37.77 | 18.84 | 2.00 | 0.0246 | 212 | 1 |
| NM_001349451 | SETD5 | 14.06 | 5.62 | 2.50 | 0.0499 | 146 | 1 |
| NM_001242729 | ARHGEF38 | 20.15 | 40.55 | 0.50 | 0.0203 | 154 | 1 |
| NM_001168235 | FREM3 | 41.34 | 18.61 | 2.22 | 0.0201 | 91 | 1 |
| NM_001256449 | GUCY1A1 | 38.18 | 18.58 | 2.05 | 0.0202 | 246 | 0 |
| NM_001130685 | GUCY1A1 | 38.12 | 18.67 | 2.04 | 0.0202 | 226 | 1 |
| NM_001331024 | KLHL2 | 29.07 | 14.09 | 2.06 | 0.0445 | 102 | 1 |
| NM_001301647 | MFAP3L | 8.58 | 23.51 | 0.36 | 0.0469 | 152 | 1 |
| NM_007281 | SCRG1 | 16.26 | 33.10 | 0.49 | 0.0228 | 70 | 1 |
| NM_012318 | LETM1 | 22.04 | 7.43 | 2.97 | 0.0422 | 158 | 1 |
| NM_005245 | FAT1 | 13.94 | 30.04 | 0.46 | 0.0350 | 262 | 1 |
| NM_001085399 | RELL1 | 8.61 | 19.31 | 0.45 | 0.0389 | 240 | 1 |
| NM_014988 | LIMCH1 | 17.91 | 37.71 | 0.47 | 0.0246 | 190 | 1 |
| NM_033211 | C5orf30 | 12.86 | 32.13 | 0.40 | 0.0394 | 265 | 0 |
| NM_001316969 | C5orf30 | 11.66 | 28.23 | 0.41 | 0.0479 | 233 | 1 |
| NM_001354925 | IRF1 | 18.92 | 7.75 | 2.44 | 0.0363 | 138 | 1 |
| NM_001287583 | CDC25C | 20.14 | 8.29 | 2.43 | 0.0202 | 171 | 1 |
| NM_030571 | NDFIP1 | 31.74 | 14.34 | 2.21 | 0.0200 | 184 | 1 |
| NM_001387 | DPYSL3 | 34.49 | 16.83 | 2.05 | 0.0214 | 148 | 1 |
| NM_173465 | COL23A1 | 29.25 | 13.87 | 2.11 | 0.0171 | 18 | 1 |
| NM_001317724 | NOL7 | 26.12 | 10.91 | 2.39 | 0.0203 | 17 | 1 |
| NM_014739 | BCLAF1 | 8.80 | 18.74 | 0.47 | 0.0375 | 37 | 1 |
| NM_006734 | HIVEP2 | 24.56 | 7.89 | 3.11 | 0.0171 | 208 | 1 |
| NM_001122740 | ESR1 | 15.31 | 32.24 | 0.47 | 0.0171 | 164 | 1 |
| NM_005618 | DLL1 | 13.03 | 29.18 | 0.45 | 0.0203 | 213 | 1 |
| NM_001286379 | FAM120B | 12.09 | 27.61 | 0.44 | 0.0203 | 216 | 0 |
| NM_001354934 | RIPK1 | 10.40 | 22.79 | 0.46 | 0.0375 | 15 | 1 |
| NM_003192 | TBCC | 19.16 | 7.57 | 2.53 | 0.0244 | 198 | 1 |
| NM_001137560 | TMEM151B | 27.68 | 13.62 | 2.03 | 0.0256 | 169 | 1 |
| NM_152731 | BEND6 | 32.57 | 12.91 | 2.52 | 0.0493 | 122 | 1 |
| NM_002395 | ME1 | 18.21 | 38.17 | 0.48 | 0.0163 | 177 | 1 |
| NM_001302621 | ACHE | 33.77 | 11.10 | 3.04 | 0.0182 | 197 | 1 |
| NM_001302622 | ACHE | 30.37 | 11.10 | 2.74 | 0.0201 | 255 | 0 |
| NM_001331192 | SVOPL | 12.53 | 27.71 | 0.45 | 0.0435 | 236 | 1 |
| NM_019015 | CHPF2 | 9.67 | 25.90 | 0.37 | 0.0208 | 69 | 1 |
| NM_003930 | SKAP2 | 7.45 | 20.81 | 0.36 | 0.0281 | 168 | 1 |
| NM_002541 | OGDH | 19.90 | 9.58 | 2.08 | 0.0457 | 86 | 1 |
| NM_031449 | ZMIZ2 | 21.60 | 10.14 | 2.13 | 0.0272 | 143 | 1 |
| NM_003088 | FSCN1 | 28.36 | 13.01 | 2.18 | 0.0218 | 38 | 1 |
| NM_145111 | FAM200A | 23.54 | 10.08 | 2.33 | 0.0191 | 126 | 1 |
| NM_001284308 | ADAP1 | 11.20 | 22.81 | 0.49 | 0.0214 | 173 | 1 |
| NM_014943 | ZHX2 | 11.49 | 24.78 | 0.46 | 0.0171 | 97 | 1 |
| NM_145003 | TSNARE1 | 30.87 | 11.15 | 2.77 | 0.0290 | 80 | 1 |
| NM_201380 | PLEC | 10.52 | 21.88 | 0.48 | 0.0352 | 79 | 1 |
| NM_003841 | TNFRSF10C | 40.63 | 19.82 | 2.05 | 0.0332 | 51 | 1 |
| NM_025115 | TTI2 | 13.75 | 31.58 | 0.44 | 0.0163 | 273 | 0 |
| NM_001330505 | TTI2 | 13.75 | 31.04 | 0.44 | 0.0163 | 271 | 1 |
| NM_001279360 | LYPLA1 | 9.33 | 19.03 | 0.49 | 0.0201 | 179 | 1 |
| NM_015404 | WHRN | 13.51 | 28.71 | 0.47 | 0.0428 | 107 | 1 |
| NM_001199233 | TEX48 | 11.50 | 26.97 | 0.43 | 0.0182 | 130 | 1 |
| NM_001353054 | GSN | 19.08 | 8.68 | 2.20 | 0.0481 | 75 | 1 |
| NM_001353069 | GSN | 11.91 | 28.63 | 0.42 | 0.0208 | 132 | 1 |
| NM_018387 | STRBP | 7.92 | 22.70 | 0.35 | 0.0163 | 2 | 0 |
| NM_001171137 | STRBP | 7.92 | 22.70 | 0.35 | 0.0163 | 1 | 1 |
| NM_005347 | HSPA5 | 16.89 | 4.43 | 3.81 | 0.0201 | 257 | 1 |
| NM_001287046 | SH3GLB2 | 28.61 | 12.21 | 2.34 | 0.0201 | 25 | 1 |
| NM_014286 | NCS1 | 31.37 | 13.92 | 2.25 | 0.0203 | 22 | 1 |
| NM_001282957 | CFAP77 | 17.59 | 38.28 | 0.46 | 0.0163 | 230 | 1 |
| NM_001162427 | TSC1 | 10.44 | 22.92 | 0.46 | 0.0339 | 275 | 1 |
| NM_001080849 | DNLZ | 7.23 | 21.43 | 0.34 | 0.0167 | 206 | 1 |
| NM_001287033 | STOML2 | 11.74 | 25.97 | 0.45 | 0.0214 | 239 | 1 |
| NM_053067 | UBQLN1 | 22.92 | 10.72 | 2.14 | 0.0479 | 96 | 1 |
| NM_001330701 | AGTPBP1 | 8.19 | 22.21 | 0.37 | 0.0244 | 64 | 1 |
| NM_153698 | AAED1 | 29.10 | 12.58 | 2.31 | 0.0167 | 141 | 1 |

FDR was calculated using the Benjamini-Hochberg procedure. The gene rank was calculated by sigFeature. In the Retain column, 1 and 0 indicate whether the gene retains in 228 genes.
