# Supplementary material for: Genome-wide nucleosome footprints of plasma cfDNA predict preterm birth: A case-control study
Source: PLoS Med. 2025 Apr 15;22(4):e1004571. doi: 10.1371/journal.pmed.1004571 (PMC11999135; doi:10.1371/journal.pmed.1004571)
Supplement: S6 Table — (DOCX) [file pmed.1004571.s013.docx]

**S6 Table. Correlation coefficients of the filtered genes.**

| RefSeq_Name1 | RefSeq_Name1 | Coefficient |
| --- | --- | --- |
| NM_001258371 | NM_001009899 | -0.2630 |
| NM_153347 | NM_001320410 | -0.2624 |
| NM_001104 | NM_001009899 | -0.2608 |
| NM_006897 | NM_001300815 | -0.2594 |
| NM_006897 | NM_001280 | -0.2594 |
| NM_001320193 | NM_001135734 | -0.2564 |
| NM_001113201 | NM_001135734 | -0.2564 |
| NM_001242413 | NM_003930 | -0.2543 |
| NM_023009 | NM_001142544 | -0.2537 |
| NM_138396 | NM_001288951 | -0.2524 |
| NM_005608 | NM_018387 | -0.2521 |
| NM_001198983 | NM_031449 | -0.2512 |
| NM_014824 | NM_001199233 | -0.2512 |
| NM_001040153 | NM_001350062 | -0.2504 |
| NM_001278575 | NM_005862 | -0.2496 |
| NM_005608 | NM_001171137 | -0.2488 |
| NM_001258289 | NM_001301647 | -0.2440 |
| NM_001278575 | NM_001080400 | -0.2433 |
| NM_001352019 | NM_015404 | -0.2425 |
| NM_007281 | NM_031449 | -0.2415 |
| NM_020759 | NM_001200018 | -0.2407 |
| NM_001008409 | NM_001287046 | -0.2407 |
| NM_138705 | NM_005248 | -0.2388 |
| NM_001166007 | NM_001142544 | -0.2387 |
| NM_003594 | NM_001080400 | -0.2383 |
| NM_001009899 | NM_001355243 | -0.2382 |
| NM_182565 | NM_001168335 | -0.2377 |
| NM_020759 | NM_001200029 | -0.2369 |
| NM_001104 | NM_001008779 | -0.2368 |
| NM_001009899 | NM_007362 | -0.2365 |
| NM_001267728 | NM_001142448 | -0.2363 |
| NM_019020 | NM_018155 | -0.2355 |
| NM_001297643 | NM_002673 | -0.2339 |
| NM_001277333 | NM_001302621 | -0.2320 |
| NM_021912 | NM_145003 | -0.2293 |
| NM_015205 | NM_020759 | -0.2288 |
| NM_005985 | NM_152731 | -0.2277 |
| NM_024671 | NM_003841 | -0.2275 |
| NM_001135734 | NM_001308036 | -0.2246 |
| NM_001142448 | NM_002673 | -0.2238 |
| NM_001272042 | NM_203351 | -0.2236 |
| NM_182704 | NM_001256449 | -0.2235 |
| NM_001030287 | NM_005248 | -0.2223 |
| NM_002809 | NM_001355243 | -0.2221 |
| NM_001080414 | NM_003250 | -0.2218 |
| NM_130434 | NM_030571 | -0.2215 |
| NM_001040153 | NM_000104 | -0.2212 |
| NM_001278575 | NM_001330519 | -0.2208 |
| NM_020692 | NM_001278575 | -0.2208 |
| NM_182704 | NM_001009899 | -0.2203 |
| NM_001277333 | NM_001302622 | -0.2196 |
| NM_024297 | NM_001282972 | -0.2196 |
| NM_018398 | NM_001331192 | -0.2195 |
| NM_020692 | NM_145640 | -0.2192 |
| NM_004386 | NM_001042519 | -0.2187 |
| NM_001271983 | NM_021824 | -0.2186 |
| NM_004386 | NM_152731 | -0.2161 |
| NM_001242413 | NM_001286679 | -0.2159 |
| NM_001030287 | NM_000891 | -0.2157 |
| NM_002541 | NM_001353069 | -0.2157 |
| NM_015205 | NM_001349290 | -0.2156 |
| NM_024671 | NM_001008779 | -0.2155 |
| NM_001323265 | NM_003930 | -0.2155 |
| NM_001136505 | NM_145003 | -0.2152 |
| NM_002809 | NM_007362 | -0.2143 |
| NM_024297 | NM_005336 | -0.2142 |
| NM_007038 | NM_003392 | -0.2141 |
| NM_004386 | NM_032321 | -0.2133 |
| NM_001136505 | NM_001349622 | -0.2131 |
| NM_001136505 | NM_001349618 | -0.2131 |
| NM_016333 | NM_001243702 | -0.2130 |
| NM_152716 | NM_001350062 | -0.2127 |
| NM_182704 | NM_001130685 | -0.2122 |
| NM_001136505 | NM_001349626 | -0.2118 |
| NM_001134877 | NM_031449 | -0.2111 |
| NM_001136505 | NM_001349616 | -0.2108 |
| NM_001272042 | NM_001330431 | -0.2106 |
| NM_001136505 | NM_001349615 | -0.2104 |
| NM_001271983 | NM_001142355 | -0.2102 |
| NM_001136505 | NM_001349619 | -0.2100 |
| NM_001277075 | NM_080622 | -0.2096 |
| NM_003268 | NM_001256160 | -0.2096 |
| NM_001286094 | NM_000104 | -0.2093 |
| NM_001291281 | NM_005862 | -0.2089 |
| NM_203351 | NM_001282957 | -0.2083 |
| NM_182507 | NM_015205 | -0.2083 |
| NM_001171815 | NM_001278575 | -0.2082 |
| NM_001286094 | NM_001353054 | -0.2081 |
| NM_001323600 | NM_021141 | -0.2078 |
| NM_015205 | NM_005336 | -0.2067 |
| NM_006912 | NM_014430 | -0.2065 |
| NM_001286094 | NM_001040715 | -0.2062 |
| NM_001040153 | NM_001242474 | -0.2058 |
| NM_153321 | NM_001256449 | -0.2055 |
| NM_001143769 | NM_001302621 | -0.2054 |
| NM_005248 | NM_153321 | -0.2053 |
| NM_033400 | NM_003392 | -0.2051 |
| NM_001135734 | NM_004920 | -0.2051 |
| NM_001025249 | NM_003841 | -0.2050 |
| NM_001195259 | NM_001008779 | -0.2049 |
| NM_178841 | NM_001085399 | -0.2046 |
| NM_001104 | NM_001040153 | -0.2044 |
| NM_002809 | NM_001308036 | -0.2043 |
| NM_001316935 | NM_001199233 | -0.2039 |
| NM_030573 | NM_005347 | -0.2038 |
| NM_001267728 | NM_001350062 | -0.2037 |
| NM_020770 | NM_001279360 | -0.2026 |
| NM_001206878 | NM_003930 | -0.2025 |
| NM_001258371 | NM_002738 | -0.2022 |
| NM_007129 | NM_001162427 | -0.2022 |
| NM_020437 | NM_001171137 | -0.2020 |
| NM_001267728 | NM_012158 | -0.2018 |
| NM_033631 | NM_001328666 | -0.2015 |
| NM_006912 | NM_001318807 | -0.2012 |
| NM_001320193 | NM_001330505 | -0.2009 |
| NM_032321 | NM_002213 | -0.2008 |
| NM_001278631 | NM_145003 | -0.2007 |
| NM_001330431 | NM_001282957 | -0.2006 |
| NM_001168335 | NM_001200018 | -0.2003 |
| NM_006897 | NM_004920 | -0.2002 |
| NM_001030287 | NM_021824 | -0.2000 |
| NM_001258289 | NM_178439 | 0.2001 |
| NM_001330431 | NM_014286 | 0.2002 |
| NM_001142315 | NM_003869 | 0.2005 |
| NM_153347 | NM_001308036 | 0.2006 |
| NM_182507 | NM_001349290 | 0.2008 |
| NM_005426 | NM_001168335 | 0.2008 |
| NM_016333 | NM_006270 | 0.2008 |
| NM_001318807 | NM_004161 | 0.2009 |
| NM_001258289 | NM_006897 | 0.2009 |
| NM_017643 | NM_002395 | 0.2010 |
| NM_001352019 | NM_000104 | 0.2011 |
| NM_032701 | NM_020459 | 0.2014 |
| NM_001302622 | NM_145003 | 0.2015 |
| NM_001331024 | NM_001302622 | 0.2015 |
| NM_001352318 | NM_001130685 | 0.2015 |
| NM_006912 | NM_001166007 | 0.2015 |
| NM_001258289 | NM_001302622 | 0.2016 |
| NM_003174 | NM_178841 | 0.2017 |
| NM_007129 | NM_001146345 | 0.2017 |
| NM_006897 | NM_001330701 | 0.2020 |
| NM_005537 | NM_001349451 | 0.2020 |
| NM_003098 | NM_001354925 | 0.2022 |
| NM_001297643 | NM_001278631 | 0.2022 |
| NM_001297643 | NM_019015 | 0.2025 |
| NM_001317724 | NM_001302622 | 0.2026 |
| NM_001009899 | NM_001130685 | 0.2027 |
| NM_134324 | NM_001350062 | 0.2029 |
| NM_005248 | NM_001271611 | 0.2029 |
| NM_005608 | NM_001349451 | 0.2031 |
| NM_001258222 | NM_000104 | 0.2031 |
| NM_002738 | NM_001040715 | 0.2031 |
| NM_003268 | NM_001328666 | 0.2032 |
| NM_001136505 | NM_007038 | 0.2033 |
| NM_003098 | NM_001331024 | 0.2033 |
| NM_020719 | NM_003088 | 0.2034 |
| NM_007129 | NM_001287583 | 0.2034 |
| NM_001190919 | NM_003392 | 0.2036 |
| NM_018155 | NM_001282957 | 0.2037 |
| NM_001104 | NM_007038 | 0.2037 |
| NM_001256405 | NM_001302621 | 0.2041 |
| NM_001278575 | NM_001321691 | 0.2044 |
| NM_153698 | NM_080821 | 0.2044 |
| NM_001316994 | NM_014286 | 0.2046 |
| NM_001318507 | NM_001313904 | 0.2046 |
| NM_005537 | NM_002673 | 0.2047 |
| NM_001002031 | NM_014430 | 0.2047 |
| NM_025209 | NM_024671 | 0.2049 |
| NM_001142315 | NM_001012334 | 0.2049 |
| NM_015404 | NM_001287033 | 0.2050 |
| NM_000891 | NM_001146345 | 0.2053 |
| NM_001242474 | NM_001042519 | 0.2054 |
| NM_005426 | NM_001349742 | 0.2055 |
| NM_001143769 | NM_006734 | 0.2055 |
| NM_001009899 | NM_001287583 | 0.2057 |
| NM_001242800 | NM_032321 | 0.2060 |
| NM_001323265 | NM_001098576 | 0.2060 |
| NM_001330505 | NM_001330701 | 0.2060 |
| NM_006912 | NM_005475 | 0.2061 |
| NM_003250 | NM_001354925 | 0.2062 |
| NM_001098576 | NM_001278631 | 0.2062 |
| NM_001271983 | NM_001278575 | 0.2064 |
| NM_006270 | NM_001302622 | 0.2064 |
| NM_001323600 | NM_003250 | 0.2064 |
| NM_005608 | NM_001243702 | 0.2067 |
| NM_004161 | NM_032319 | 0.2068 |
| NM_005475 | NM_203351 | 0.2070 |
| NM_001271611 | NM_001349279 | 0.2071 |
| NM_001318170 | NM_145003 | 0.2073 |
| NM_001286679 | NM_152362 | 0.2074 |
| NM_203351 | NM_014286 | 0.2074 |
| NM_001271611 | NM_001200029 | 0.2076 |
| NM_005862 | NM_001287033 | 0.2077 |
| NM_021141 | NM_018398 | 0.2078 |
| NM_023009 | NM_001242474 | 0.2081 |
| NM_001009899 | NM_001256449 | 0.2083 |
| NM_207340 | NM_002213 | 0.2087 |
| NM_007038 | NM_001355243 | 0.2089 |
| NM_001080414 | NM_001349290 | 0.2092 |
| NM_003174 | NM_001171815 | 0.2092 |
| NM_001300815 | NM_201380 | 0.2092 |
| NM_001280 | NM_201380 | 0.2092 |
| NM_004461 | NM_032319 | 0.2093 |
| NM_182704 | NM_007038 | 0.2094 |
| NM_018030 | NM_001195259 | 0.2095 |
| NM_003098 | NM_001282957 | 0.2102 |
| NM_032319 | NM_001200018 | 0.2103 |
| NM_001300815 | NM_001349279 | 0.2108 |
| NM_001280 | NM_001349279 | 0.2108 |
| NM_178511 | NM_001008409 | 0.2112 |
| NM_001143769 | NM_004386 | 0.2113 |
| NM_134324 | NM_015404 | 0.2113 |
| NM_020759 | NM_001277333 | 0.2113 |
| NM_001331024 | NM_001302621 | 0.2113 |
| NM_001291281 | NM_001142544 | 0.2116 |
| NM_005248 | NM_004907 | 0.2117 |
| NM_021141 | NM_001302621 | 0.2117 |
| NM_025115 | NM_001330701 | 0.2118 |
| NM_024671 | NM_006270 | 0.2119 |
| NM_001349451 | NM_053067 | 0.2119 |
| NM_001200018 | NM_001287046 | 0.2119 |
| NM_001008409 | NM_001302621 | 0.2120 |
| NM_001113201 | NM_007038 | 0.2123 |
| NM_001190919 | NM_202001 | 0.2123 |
| NM_001324494 | NM_001352278 | 0.2125 |
| NM_001198965 | NM_001349451 | 0.2126 |
| NM_004161 | NM_014943 | 0.2127 |
| NM_012158 | NM_001080414 | 0.2128 |
| NM_001025249 | NM_001349616 | 0.2128 |
| NM_006690 | NM_001353069 | 0.2131 |
| NM_182507 | NM_005336 | 0.2134 |
| NM_022482 | NM_003088 | 0.2135 |
| NM_020889 | NM_001080849 | 0.2136 |
| NM_001040715 | NM_006734 | 0.2136 |
| NM_001002031 | NM_001318807 | 0.2136 |
| NM_003594 | NM_021912 | 0.2139 |
| NM_003392 | NM_003841 | 0.2140 |
| NM_001042519 | NM_001287583 | 0.2141 |
| NM_001193611 | NM_001279360 | 0.2142 |
| NM_152716 | NM_001098576 | 0.2143 |
| NM_001258222 | NM_080821 | 0.2144 |
| NM_001352318 | NM_001256449 | 0.2145 |
| NM_001171815 | NM_003841 | 0.2146 |
| NM_001025249 | NM_001349615 | 0.2146 |
| NM_002395 | NM_001162427 | 0.2146 |
| NM_001025249 | NM_001349619 | 0.2146 |
| NM_017516 | NM_001009899 | 0.2147 |
| NM_004920 | NM_018398 | 0.2147 |
| NM_001025249 | NM_001349626 | 0.2148 |
| NM_001025249 | NM_001349622 | 0.2150 |
| NM_005475 | NM_003869 | 0.2152 |
| NM_001200029 | NM_001287046 | 0.2152 |
| NM_005537 | NM_130434 | 0.2155 |
| NM_003419 | NM_003930 | 0.2155 |
| NM_003174 | NM_003250 | 0.2158 |
| NM_001171815 | NM_001313904 | 0.2161 |
| NM_007038 | NM_001286379 | 0.2166 |
| NM_022482 | NM_003098 | 0.2169 |
| NM_024297 | NM_003841 | 0.2170 |
| NM_001313904 | NM_001308036 | 0.2174 |
| NM_014430 | NM_014943 | 0.2174 |
| NM_014587 | NM_178511 | 0.2174 |
| NM_005537 | NM_001137560 | 0.2175 |
| NM_001352318 | NM_005347 | 0.2177 |
| NM_005608 | NM_005248 | 0.2178 |
| NM_001271611 | NM_001200018 | 0.2179 |
| NM_153698 | NM_005248 | 0.2180 |
| NM_007038 | NM_005618 | 0.2182 |
| NM_001030287 | NM_025115 | 0.2182 |
| NM_001025249 | NM_014306 | 0.2183 |
| NM_001206878 | NM_001354925 | 0.2183 |
| NM_004907 | NM_001349279 | 0.2184 |
| NM_001258222 | NM_020459 | 0.2185 |
| NM_001025249 | NM_001349618 | 0.2185 |
| NM_153347 | NM_014286 | 0.2186 |
| NM_001098576 | NM_001320410 | 0.2189 |
| NM_001349279 | NM_001256449 | 0.2189 |
| NM_001166007 | NM_001321691 | 0.2191 |
| NM_001242474 | NM_032321 | 0.2198 |
| NM_032319 | NM_001200029 | 0.2200 |
| NM_000104 | NM_002213 | 0.2203 |
| NM_006912 | NM_021141 | 0.2211 |
| NM_004907 | NM_001200029 | 0.2211 |
| NM_001137560 | NM_014286 | 0.2211 |
| NM_001128855 | NM_014306 | 0.2213 |
| NM_001272042 | NM_001279360 | 0.2214 |
| NM_001080400 | NM_152731 | 0.2216 |
| NM_005426 | NM_002738 | 0.2217 |
| NM_001349618 | NM_001195259 | 0.2218 |
| NM_004386 | NM_001282972 | 0.2219 |
| NM_006897 | NM_001040153 | 0.2224 |
| NM_001349616 | NM_001195259 | 0.2227 |
| NM_001349626 | NM_001195259 | 0.2227 |
| NM_005248 | NM_001287583 | 0.2228 |
| NM_004386 | NM_005336 | 0.2229 |
| NM_001349279 | NM_001130685 | 0.2230 |
| NM_134324 | NM_001136505 | 0.2234 |
| NM_152450 | NM_153321 | 0.2234 |
| NM_001349622 | NM_014306 | 0.2235 |
| NM_001256160 | NM_001349451 | 0.2236 |
| NM_178841 | NM_006164 | 0.2236 |
| NM_001349615 | NM_001195259 | 0.2239 |
| NM_021912 | NM_005862 | 0.2241 |
| NM_178841 | NM_001387 | 0.2243 |
| NM_001171815 | NM_006164 | 0.2244 |
| NM_001030287 | NM_001330505 | 0.2245 |
| NM_003174 | NM_001206878 | 0.2247 |
| NM_001008779 | NM_001387 | 0.2250 |
| NM_001349619 | NM_001195259 | 0.2250 |
| NM_178841 | NM_001313904 | 0.2250 |
| NM_001349619 | NM_014306 | 0.2253 |
| NM_134324 | NM_002213 | 0.2255 |
| NM_004907 | NM_020437 | 0.2257 |
| NM_001098576 | NM_021912 | 0.2259 |
| NM_152716 | NM_000891 | 0.2260 |
| NM_001098206 | NM_001291281 | 0.2263 |
| NM_001349616 | NM_014306 | 0.2264 |
| NM_001349615 | NM_014306 | 0.2264 |
| NM_001349626 | NM_014306 | 0.2268 |
| NM_004161 | NM_001080849 | 0.2268 |
| NM_001323600 | NM_001206878 | 0.2275 |
| NM_014988 | NM_033211 | 0.2275 |
| NM_001297643 | NM_014988 | 0.2277 |
| NM_153347 | NM_203351 | 0.2280 |
| NM_003250 | NM_001200029 | 0.2280 |
| NM_001085399 | NM_005347 | 0.2281 |
| NM_001349622 | NM_001195259 | 0.2285 |
| NM_023009 | NM_015404 | 0.2286 |
| NM_001206878 | NM_003841 | 0.2289 |
| NM_001277333 | NM_001330701 | 0.2291 |
| NM_001352322 | NM_005347 | 0.2301 |
| NM_001349618 | NM_014306 | 0.2301 |
| NM_001318807 | NM_014943 | 0.2306 |
| NM_001349279 | NM_201380 | 0.5306 |
| NM_004907 | NM_001200018 | 0.2318 |
| NM_003594 | NM_001278631 | 0.2319 |
| NM_152716 | NM_001146345 | 0.2320 |
| NM_001206878 | NM_001355243 | 0.2321 |
| NM_001008409 | NM_001302622 | 0.2321 |
| NM_001352322 | NM_001130685 | 0.2322 |
| NM_001313904 | NM_007362 | 0.2324 |
| NM_001207068 | NM_001085399 | 0.2325 |
| NM_005248 | NM_052940 | 0.2333 |
| NM_153347 | NM_001330431 | 0.2335 |
| NM_018398 | NM_001387 | 0.2340 |
| NM_001318507 | NM_001387 | 0.2342 |
| NM_001352322 | NM_001256449 | 0.2350 |
| NM_022041 | NM_021248 | 0.2350 |
| NM_001242413 | NM_001206878 | 0.2352 |
| NM_001286679 | NM_001128855 | 0.2353 |
| NM_001267728 | NM_001256160 | 0.2358 |
| NM_001171815 | NM_001387 | 0.2372 |
| NM_001297643 | NM_014739 | 0.2381 |
| NM_005248 | NM_001008779 | 0.2383 |
| NM_000969 | NM_014286 | 0.2383 |
| NM_001012334 | NM_001287583 | 0.2385 |
| NM_006270 | NM_001302621 | 0.2395 |
| NM_001313904 | NM_001355243 | 0.2400 |
| NM_001012334 | NM_001130685 | 0.2401 |
| NM_021912 | NM_001316994 | 0.2403 |
| NM_001271611 | NM_020437 | 0.2403 |
| NM_002738 | NM_005245 | 0.2407 |
| NM_032321 | NM_001287583 | 0.2418 |
| NM_005248 | NM_001316935 | 0.2422 |
| NM_003250 | NM_001200018 | 0.2423 |
| NM_022041 | NM_002395 | 0.2425 |
| NM_134324 | NM_020529 | 0.2431 |
| NM_001025249 | NM_005862 | 0.2432 |
| NM_001271983 | NM_001122740 | 0.2433 |
| NM_001242413 | NM_001098576 | 0.2442 |
| NM_014306 | NM_001288953 | 0.2454 |
| NM_202001 | NM_018398 | 0.2457 |
| NM_001025249 | NM_001080849 | 0.2458 |
| NM_001242413 | NM_031449 | 0.2459 |
| NM_001300815 | NM_001195259 | 0.2467 |
| NM_001280 | NM_001195259 | 0.2467 |
| NM_001012334 | NM_001256449 | 0.2468 |
| NM_001318170 | NM_001331024 | 0.2470 |
| NM_020529 | NM_002213 | 0.2475 |
| NM_130434 | NM_080821 | 0.2476 |
| NM_001256160 | NM_001168235 | 0.2481 |
| NM_001320410 | NM_003098 | 0.2485 |
| NM_001206878 | NM_007362 | 0.2490 |
| NM_152362 | NM_002395 | 0.2496 |
| NM_001316935 | NM_000891 | 0.2498 |
| NM_005608 | NM_005537 | 0.2510 |
| NM_178841 | NM_001008779 | 0.2534 |
| NM_001320410 | NM_001085399 | 0.2538 |
| NM_001171815 | NM_001008779 | 0.2541 |
| NM_052940 | NM_000891 | 0.2544 |
| NM_152450 | NM_001331192 | 0.2558 |
| NM_001080400 | NM_000104 | 0.2561 |
| NM_001080400 | NM_001349315 | 0.2571 |
| NM_001323600 | NM_001354690 | 0.2594 |
| NM_001207069 | NM_001085399 | 0.2599 |
| NM_002213 | NM_001330505 | 0.2604 |
| NM_003566 | NM_014306 | 0.2605 |
| NM_001318507 | NM_001008779 | 0.2626 |
| NM_001002031 | NM_001040153 | 0.2645 |
| NM_019020 | NM_001316994 | 0.2648 |
| NM_002213 | NM_025115 | 0.2653 |
| NM_001349451 | NM_001256449 | 0.2662 |
| NM_001267728 | NM_001349451 | 0.2666 |
| NM_001349451 | NM_001130685 | 0.2667 |
| NM_003174 | NM_001354690 | 0.2678 |
| NM_000104 | NM_001162427 | 0.2684 |
| NM_005426 | NM_001243702 | 0.2736 |
| NM_000891 | NM_003088 | 0.2752 |
| NM_001206878 | NM_001308036 | 0.2757 |
| NM_153698 | NM_005608 | 0.2812 |
| NM_153347 | NM_001352322 | 0.2814 |
| NM_153347 | NM_001352318 | 0.2819 |
| NM_001256449 | NM_145003 | 0.2903 |
| NM_001321691 | NM_001085399 | 0.2918 |
| NM_003098 | NM_001085399 | 0.2918 |
| NM_016062 | NM_001277333 | 0.2932 |
| NM_005537 | NM_001256160 | 0.2943 |
| NM_002809 | NM_001009899 | 0.2945 |
| NM_001130685 | NM_145003 | 0.2952 |
| NM_018030 | NM_201380 | 0.2983 |
| NM_001172646 | NM_007281 | 0.3052 |
| NM_003869 | NM_001277333 | 0.3053 |
| NM_006912 | NM_001387 | 0.3108 |
| NM_000969 | NM_001168235 | 0.3108 |
| NM_020759 | NM_001330701 | 0.3193 |
| NM_052940 | NM_020437 | 0.3262 |
| NM_001316935 | NM_020437 | 0.3276 |
| NM_001025249 | NM_003098 | 0.3419 |
| NM_001320410 | NM_001025249 | 0.3433 |
| NM_001080414 | NM_020759 | 0.3502 |
| NM_001025249 | NM_001085399 | 0.3845 |
| NM_001349315 | NM_001349290 | 0.4018 |
| NM_001349315 | NM_005336 | 0.5170 |
| NM_001349315 | NM_001282972 | 0.5183 |
| NM_001313904 | NM_006164 | 0.6030 |
| NM_207340 | NM_001104 | 0.6722 |
| NM_001190919 | NM_003250 | 0.6884 |
| NM_003419 | NM_001242474 | 0.6992 |
| NM_005537 | NM_001267728 | 0.7083 |
| NM_001278631 | NM_021912 | 0.7251 |
| NM_003419 | NM_001242800 | 0.7475 |
| NM_001258371 | NM_001104 | 0.7831 |
| NM_001321691 | NM_001207068 | 0.7887 |
| NM_005336 | NM_001349290 | 0.8009 |
| NM_001282972 | NM_001349290 | 0.8022 |
| NM_033211 | NM_001316969 | 0.8453 |
| NM_001321691 | NM_001207069 | 0.8492 |
| NM_001113201 | NM_001320193 | 0.8591 |
| NM_001270550 | NM_001012334 | 0.8657 |
| NM_001198983 | NM_001134877 | 0.8850 |
| NM_130906 | NM_021824 | 0.8880 |
| NM_001352019 | NM_014587 | 0.8899 |
| NM_001308036 | NM_001355243 | 0.9011 |
| NM_001242413 | NM_001323265 | 0.9042 |
| NM_001352322 | NM_001352318 | 0.9067 |
| NM_130906 | NM_001142355 | 0.9113 |
| NM_207340 | NM_001258371 | 0.9139 |
| NM_001308036 | NM_007362 | 0.9149 |
| NM_001193611 | NM_001272042 | 0.9282 |
| NM_001207069 | NM_001207068 | 0.9397 |
| NM_003174 | NM_001323600 | 0.9546 |
| NM_001350765 | NM_001256875 | 0.9586 |
| NM_001302621 | NM_001302622 | 0.9594 |
| NM_001042519 | NM_032321 | 0.9608 |
| NM_001242800 | NM_001242474 | 0.9619 |
| NM_005618 | NM_001286379 | 0.9632 |
| NM_001142355 | NM_021824 | 0.9729 |
| NM_001171815 | NM_001318507 | 0.9767 |
| NM_001271611 | NM_004907 | 0.9809 |
| NM_001014283 | NM_001349742 | 0.9833 |
| NM_001349618 | NM_001349622 | 0.9848 |
| NM_001330431 | NM_203351 | 0.9861 |
| NM_001349618 | NM_001349616 | 0.9870 |
| NM_007362 | NM_001355243 | 0.9870 |
| NM_001349618 | NM_001349619 | 0.9876 |
| NM_001349618 | NM_001349615 | 0.9877 |
| NM_001171815 | NM_178841 | 0.9883 |
| NM_016062 | NM_003869 | 0.9886 |
| NM_001349618 | NM_001349626 | 0.9897 |
| NM_178841 | NM_001318507 | 0.9906 |
| NM_001256449 | NM_001130685 | 0.9910 |
| NM_014430 | NM_001318807 | 0.9925 |
| NM_001200029 | NM_001200018 | 0.9928 |
| NM_001349626 | NM_001349622 | 0.9951 |
| NM_018387 | NM_001171137 | 0.9958 |
| NM_005336 | NM_001282972 | 0.9966 |
| NM_001349615 | NM_001349622 | 0.9970 |
| NM_001349619 | NM_001349622 | 0.9972 |
| NM_001349626 | NM_001349616 | 0.9972 |
| NM_001316935 | NM_052940 | 0.9972 |
| NM_001349616 | NM_001349622 | 0.9979 |
| NM_001349626 | NM_001349619 | 0.9979 |
| NM_001349626 | NM_001349615 | 0.9981 |
| NM_033419 | NM_001289936 | 0.9981 |
| NM_025115 | NM_001330505 | 0.9982 |
| NM_001349615 | NM_001349616 | 0.9992 |
| NM_001349619 | NM_001349616 | 0.9993 |
| NM_001349615 | NM_001349619 | 0.9999 |
| NM_001300815 | NM_001280 | 1.0000 |

The coefficients were calculated using the Pearson correlation coefficient.
