# Supplementary material for: Genome-wide nucleosome footprints of plasma cfDNA predict preterm birth: A case-control study
Source: PLoS Med. 2025 Apr 15;22(4):e1004571. doi: 10.1371/journal.pmed.1004571 (PMC11999135; doi:10.1371/journal.pmed.1004571)
Supplement: S7 Table — (DOCX) [file pmed.1004571.s014.docx]

**S7 Table. The genes in PTerm model**

| Refseq | Gene_symbol | Strand | Threshold |
| --- | --- | --- | --- |
| NM_001242413 | PRKCQ | - | 13.691 |
| NM_001142315 | LMO2 | - | 11.913 |
| NM_001258289 | SELENBP1 | - | 23.096 |
| NM_001010979 | C1orf189 | - | 29.195 |
| NM_006912 | RIT1 | - | 19.430 |
| NM_152716 | PATL1 | - | 12.316 |
| NM_005608 | PTPRCAP | - | 21.784 |
| NM_212469 | CHKA | - | 12.207 |
| NM_001286094 | TMEM9B | - | 11.965 |
| NM_134324 | TARBP2 | + | 11.509 |
| NM_001113201 | NACA | - | 12.787 |
| NM_005248 | FGR | - | 20.748 |
| NM_007129 | ZIC2 | + | 12.246 |
| NM_018134 | IQCC | + | 19.958 |
| NM_001198983 | TEDC1 | + | 11.267 |
| NM_033400 | ZFHX2 | - | 11.096 |
| NM_001318807 | CIDEB | - | 19.538 |
| NM_001198965 | NFATC4 | + | 15.874 |
| NM_020529 | NFKBIA | - | 11.045 |
| NM_020692 | GALNT16 | + | 24.386 |
| NM_001286679 | LARP6 | - | 22.253 |
| NM_014587 | SOX8 | + | 12.714 |
| NM_024671 | ZNF768 | - | 13.031 |
| NM_001352278 | RHOT2 | + | 10.803 |
| NM_001289936 | ERBB2 | + | 21.225 |
| NM_002809 | PSMD3 | + | 12.107 |
| NM_003250 | THRA | + | 11.108 |
| NM_017643 | MBTD1 | - | 11.211 |
| NM_001272042 | RPS6KB1 | + | 19.351 |
| NM_203351 | MAP3K3 | + | 11.200 |
| NM_000891 | KCNJ2 | + | 18.148 |
| NM_201566 | SLC16A13 | + | 20.645 |
| NM_024297 | PHF23 | - | 0.969 |
| NM_182565 | UBALD2 | + | 11.077 |
| NM_019020 | TBC1D16 | - | 14.851 |
| NM_001258222 | KCTD1 | - | 22.712 |
| NM_001168335 | ME2 | + | 12.157 |
| NM_004907 | IER2 | + | 7.960 |
| NM_001242474 | ZNF345 | + | 12.207 |
| NM_182704 | SELENOV | + | 24.065 |
| NM_006270 | RRAS | - | 1.989 |
| NM_001316994 | SPACA6 | + | 12.903 |
| NM_001195259 | TGFBR3L | + | 15.326 |
| NM_022482 | GZF1 | + | 11.883 |
| NM_001008409 | TTLL9 | + | 19.162 |
| NM_003098 | SNTA1 | - | 11.908 |
| NM_006690 | MMP24 | + | 13.761 |
| NM_021248 | CDH22 | - | 20.882 |
| NM_080821 | FAM210B | + | 23.827 |
| NM_020437 | ASPHD2 | + | 20.361 |
| NM_206895 | SNORC | + | 29.302 |
| NM_145640 | APOL3 | - | 18.118 |
| NM_001282972 | SPT7L | + | 9.734 |
| NM_001008779 | SPDYA | + | 28.058 |
| NM_000104 | CYP1B1 | - | 22.490 |
| NM_001288953 | TTC7A | + | 16.606 |
| NM_001354690 | RAF1 | - | 14.004 |
| NM_005862 | STAG1 | - | 1.101 |
| NM_018155 | SLC25A36 | + | 17.767 |
| NM_001308036 | NCBP2 | - | 12.191 |
| NM_001355243 | NCBP2-AS2 | + | 12.784 |
| NM_002673 | PLXNB1 | - | 12.437 |
| NM_001200018 | NAT6 | - | 11.979 |
| NM_001349451 | SETD5 | + | 1.137 |
| NM_001331024 | KLHL2 | + | 15.919 |
| NM_007281 | SCRG1 | - | 31.805 |
| NM_001287583 | CDC25C | - | 11.210 |
| NM_030571 | NDFIP1 | + | 23.105 |
| NM_001317724 | NOL7 | + | 12.915 |
| NM_014739 | BCLAF1 | - | 11.830 |
| NM_001354934 | RIPK1 | + | 11.870 |
| NM_019015 | CHPF2 | + | 20.677 |
| NM_003930 | SKAP2 | - | 12.562 |
| NM_002541 | OGDH | + | 11.152 |
| NM_003088 | FSCN1 | + | 11.391 |
| NM_145003 | TSNARE1 | - | 20.230 |
| NM_003841 | TNFRSF10C | + | 15.346 |
| NM_015404 | WHRN | - | 20.415 |
| NM_001353054 | GSN | + | 13.478 |
| NM_014286 | NCS1 | + | 13.691 |
| NM_001282957 | CFAP77 | + | 21.621 |
| NM_001287033 | STOML2 | - | 19.453 |
| NM_001330701 | AGTPBP1 | - | 17.448 |

Threshold means the threshold for data discretization.
