# Supplementary material for: Genome-wide nucleosome footprints of plasma cfDNA predict preterm birth: A case-control study
Source: PLoS Med. 2025 Apr 15;22(4):e1004571. doi: 10.1371/journal.pmed.1004571 (PMC11999135; doi:10.1371/journal.pmed.1004571)
Supplement: S9 Table — (DOCX) [file pmed.1004571.s015.docx]

**S9 Table.** **Performance of the optimal classifier for each model with backward and lasso algorithm**

|  | Backward | Lasso | *P*-value |
| --- | --- | --- | --- |
| SVM | | | |
| AUC (95% CI) | 0.878 (0.852-0.904) | 0.732 (0.695-0.770) | 3.6e-17 |
| LR | | | |
| AUC (95% CI) | 0.856(0.828-0.883) | 0.693(0.656-0.729) | < 2.2e-16 |
| RF | | | |
| AUC (95% CI) | 0.778(0.748-0.808) | 0.693 (0.658-0.729) | 1.4e-04 |
| XGBoost |  |  |  |
| AUC (95% CI) | 0.794 (0.761-0.828) | 0.730 (0.696-0.763) | 4.3e-04 |

The significant differences in their AUC were compared using DeLong's test. SVM=support vector machine; LR=logistic regression; RF=random forest.
