# Supplementary material for: Genome-wide nucleosome footprints of plasma cfDNA predict preterm birth: A case-control study
Source: PLoS Med. 2025 Apr 15;22(4):e1004571. doi: 10.1371/journal.pmed.1004571 (PMC11999135; doi:10.1371/journal.pmed.1004571)
Supplement: S10 Table — (DOCX) [file pmed.1004571.s016.docx]

**S10 Table. Performance of the classifiers**

| Classifier | AUC (95% CI) | Accuracy | Recall | F1-score | PPV | NPV |
| --- | --- | --- | --- | --- | --- | --- |
| SVM_Backward | 0.878(0.852-0.904) | 87.3 | 88.5 | 73.6 | 0.63 | 0.97 |
| LR_Backward | 0.856(0.828-0.883) | 84.5 | 87.4 | 69.3 | 0.57 | 0.96 |
| RF_Backward | 0.778(0.748-0.808) | 73 | 85.8 | 56.0 | 0.41 | 0.95 |
| XGBoost_Backward | 0.794 (0.761-0.828) | 79.9 | 78.7 | 61.0 | 0.50 | 0.94 |
| SVM_Lasso | 0.732(0.695-0.770) | 77.8 | 65.6 | 54.2 | 0.45 | 0.90 |
| LR_Lasso | 0.693(0.656-0.729) | 85.2 | 42.6 | 53.6 | 0.72 | 0.87 |
| RF_Lasso | 0.693 (0.658-0.729) | 65.0 | 76.5 | 46.7 | 0.34 | 0.91 |
| XGBoost_Lasso | 0.730 (0.696-0.763) | 68.2 | 80.9 | 50.4 | 0.37 | 0.93 |
| PTerm_Traning | 0.878(0.852-0.904) | 87.3 | 88.5 | 73.6 | 0.63 | 0.97 |
| PTerm_Validation1 | 0.845(0.799-0.891) | 85.8 | 82.3 | 69.9 | 0.61 | 0.95 |
| PTerm_Validation2 | 0.833(0.802-0.863) | 84.2 | 81.7 | 67.4 | 0.52 | 0.95 |
| PTerm_Validation3 | 0.812(0.761-0.863) | 80.9 | 81.7 | 63.7 | 0.57 | 0.95 |
| PTerm_All | 0.849(0.831-0.866) | 85.1 | 84.3 | 69.4 | 0.59 | 0.96 |

SVM=support vector machine; LR=logistic regression; RF=linear discriminant analysis. RF= random forest. Backward and lasso means backward and lasso feature selection.
