# Supplementary material for: Genome-wide nucleosome footprints of plasma cfDNA predict preterm birth: A case-control study
Source: PLoS Med. 2025 Apr 15;22(4):e1004571. doi: 10.1371/journal.pmed.1004571 (PMC11999135; doi:10.1371/journal.pmed.1004571)
Supplement: S11 Table — (DOCX) [file pmed.1004571.s017.docx]

**S11 Table. Comparison of the predictive efficacy of PTerm with and optimal classifiers from other models.**

| PTerm  AUC (95% CI) | Optimal classifier | AUC (95% CI) | *P*-value |
| --- | --- | --- | --- |
| 0.878 (0.852-0.904) | LR_backward | 0.856(0.828-0.883) | 0.08 |
| 0.878 (0.852-0.904) | RF_backward | 0.778(0.748-0.808) | 1.0e-06 |
| 0.878 (0.852-0.904) | XGB_backward | 0.794 (0.761-0.828) | 1.5e-04 |
| 0.878 (0.852-0.904) | SVM_lasso | 0.732(0.695-0.770) | < 2.2e-16 |
| 0.878 (0.852-0.904) | LR_lasso | 0.693(0.656-0.729) | < 2.2e-16 |
| 0.878 (0.852-0.904) | RF_lasso | 0.693 (0.658-0.729) | < 2.2e-16 |
| 0.878 (0.852-0.904) | XGB_lasso | 0.730 (0.696-0.763) | < 2.2e-16 |

PTerm was developed based on SVM model and backward feature selection method. FF=fetal fraction. SVM=support vector machine; LR=logistic regression; RF=linear discriminant analysis. RF= random forest. Backward and lasso means backward and lasso feature selection.
