# Supplementary material for: Genome-wide nucleosome footprints of plasma cfDNA predict preterm birth: A case-control study
Source: PLoS Med. 2025 Apr 15;22(4):e1004571. doi: 10.1371/journal.pmed.1004571 (PMC11999135; doi:10.1371/journal.pmed.1004571)
Supplement: S12 Table — (DOCX) [file pmed.1004571.s018.docx]

**S12 Table. The accuracy of predicting premature delivery in different birth weeks**

| Gestational weeks at birth | Sample number | Accuracy |
| --- | --- | --- |
| 28-34^+6^ | 164 | 0.866 |
| 35-36^+6^ | 354 | 0.732 |
