# Supplementary material for: Genome-wide nucleosome footprints of plasma cfDNA predict preterm birth: A case-control study
Source: PLoS Med. 2025 Apr 15;22(4):e1004571. doi: 10.1371/journal.pmed.1004571 (PMC11999135; doi:10.1371/journal.pmed.1004571)
Supplement: S13 Table — (DOCX) [file pmed.1004571.s019.docx]

**S13 Table. Functional annotation of genes in PTerm by retrieving literatures**

| Gene | Function annotation | PMID |
| --- | --- | --- |
| *ERBB2* | 1. The catalytic activity of *ErbB-2* is essential for normal embryonic development. 2. *ERBB2* gene amplification increases during the transition of proximal EGFR+ to distal HLA-G+ first trimester cell column trophoblasts. | 1. 11809799 2. 26071215 |
| *NFKBIA* | ①The expression levels of inflammation-inhibiting gene, NFKBIA, was up-regulated in the preterm pregnancies | ① 35199834 |
| *RAF1* | 1. A Novel Noonan Syndrome *RAF1* Mutation: Lethal Course in a Preterm Infant 2. *RAF1* expression is increased in myometrium with human labour and mediators of preterm labour, and inhibition of *RAF1* | 1. 26266034 2. 26811545 |
| *GSN*  (*Gelsolin*) | 1. Low plasma gelsolin levels in the first postnatal month may be associated with poor outcomes in premature infants. | ① 24862494 |
| *TMEM9B* | 1. Regulation of differentiation of Bone Marrow-Derived Mesenchymal Stem Cells | 1. 33299881 |
| *TARBP2* | 1. Significantly expressed in astrocyte maturation | ① 14703617 |
| *ZIC2* | 1. Aberrant signaling involving the *ZIC2* and *TGIF* genes are common causes of human Holoprosencephaly | ① 24753843 |
| *CYP1B1* | 1. *CYP1B1* is significantly more expressed in preterm labor patients | 1. 23916819 |
| *RPS6KB1* | 1. Deficiency of the oxidative stress–responsive kinase p70S6K1 restores autophagy and ameliorates neural tube defects in diabetic embryopathy | 1. 32416155 |
| *RIPK1* | 1. RIPK1 mRNA level was significantly increased in PE placentas. | 1. 28292463 |
| *FSCN1* | 1. miR-143 and miR-145 were significantly increased in cervical cells of women with PTB. miR-143 and miR-145 transfection decreased cervical cell number by increasing apoptosis and decreasing cell proliferation through initiation of cell cycle arrest. Cell adhesion genes, JAM-A and *FSCN1*, were downregulated with overexpression of miR-143 and miR-145. | 1. 28596604 |
| *RIT1* | 1. The mutations of *RIT1* were associated with Noonan syndrome | 1. 27109146 |
| *CHKA* | 1. Polymorphic variants of *CHKA* involved in choline pathway and the risk of intrauterine fetal death | ① 28509322 |
| *NFATC4* | 1. *NFATC4* is required for cardiac development and mitochondrial function | 1. 12750314 |
| *THRA* | 1. Termination of pregnancy with mifepristone leads to a downregulation of THRα1, THRα2 and THRβ1 in villous trophoblasts and in addition to a decreased expression of THRA in placental tissue. Decreased expression of THRα1 induced by RU486 could also be found in the decidua. | ① 26476797 |
| *MAP3K3* | 1. Appropriate activation of *MAP3K3* can play important roles in pregnancy | 1. 32175444 |
| *KCNJ2* | 1. The mutations of *KCNJ2* results in Andersen Syndrome | ① 12148092 |
| *SNTA1* | 1. *SNTA1* is associated with long-QT syndrome | ① 18591664 |
| *TTC7A* | 1. *TTC7A* Mutation in a Newborn with Multiple Intestinal Atresia and Combined Immunodeficiency | 1. 25546680 |
| *PLXNB1* | 1. A decreased expression of PLXNB1 in preeclamptic placentas may be responsible for the deficiency in Met signaling and in PE development | ① 29939944 |
| *CDC25C* | 1. The activation of the ATR-CDC25C-CDK1 pathway induces cell cycle arrest at G2-phase. | ① 28264028 |
| *SKAP2* | 1. Placental epigenome-wide association study identified loci associated with childhood Adiposity. Four candidate epigenomic regions associated with skinfold thickness, which were located within *FMN1*, *MAGI2*, *SKAP2* and *BMPR1B* genes. | 1. 33003475 |
| *OGDH* | 1. A biallelic pathogenic variant in the *OGDH* gene results in a neurological disorder with features of a mitochondrial disease. | ① 32383294 |
| *SOX8* | 1. *SOX8* takes part in mammalian testis development | ① 19647095 |
| *PSMD3* | 1. *PSMD3* takes part in hedgehog ligand biogenesis, which is crucial for the development and differentiation | ① 34674377 |
| *PRKCQ* | ①*PRKCQ* involved in the development and differentiation of early innate lymphoid progenitors | ① 29183988 |
| *CDH22* | ①*Cdh12* and *Cdh22* in the developing and adult mouse brain | 1. 20723620 |
| *SPDYA* | 1. The *SUN1*-*SPDYA* interaction plays an essential role in meiosis prophase I in cell cycle. | ① 34039995 |

PMID = PubMed Unique Identifier.
