# Supplementary material for: Genome-wide nucleosome footprints of plasma cfDNA predict preterm birth: A case-control study
Source: PLoS Med. 2025 Apr 15;22(4):e1004571. doi: 10.1371/journal.pmed.1004571 (PMC11999135; doi:10.1371/journal.pmed.1004571)
Supplement: S14 Table — (DOCX) [file pmed.1004571.s020.docx]

**S14 Table. The comparison of fetal fraction between preterm and full-term pregnancies.**

| Group | Fetal fraction of cffDNA | *P-value* |
| --- | --- | --- |
| Preterm | 0.0849 | 1 |
| Full-term | 0.0866 |  |

Two-sided Wilcoxon rank-sum test was used for the comparison of fetal fraction.
